# Branched-Chain Amino Acids Are Linked with Alzheimer’s Disease-Related Pathology and Cognitive Deficits

**DOI:** 10.3390/cells11213523

**Published:** 2022-11-07

**Authors:** Md Abu Bakkar Siddik, Caitlyn A. Mullins, Alyssa Kramer, Harsh Shah, Ritchel B. Gannaban, Masoud Zabet-Moghaddam, Ryan M. Huebinger, Vijay K. Hegde, Sheba M. J. MohanKumar, Puliyur S. MohanKumar, Andrew C. Shin

**Affiliations:** 1 Obesity and Metabolic Health Laboratory, Department of Nutritional Sciences, College of Human Sciences, Texas Tech University, Lubbock, TX 79409, USA; 2 Neurobiology of Nutrition Laboratory, Department of Nutritional Sciences, College of Human Sciences, Texas Tech University, Lubbock, TX 79409, USA; 3 Center for Biotechnology & Genomics, Texas Tech University, Lubbock, TX 79409, USA; 4 Department of Surgery, UT Southwestern Medical Center, Dallas, TX 75390, USA; 5 Department of Veterinary BioSciences and Diagnostic Imaging, College of Veterinary Medicine, University of Georgia, Athens, GA 30602, USA

**Keywords:** glucose metabolism, BCAA, neurotransmitters, Aβ-42, Tau, 5xFAD, APP/PS1, diet restriction

## Abstract

Alzheimer’s disease (AD) is an irreversible neurodegenerative disorder with a complex pathophysiology. Type 2 diabetes (T2D) is a strong risk factor for AD that shares similar abnormal features including metabolic dysregulation and brain pathology such as amyloid and/or Tau deposits. Emerging evidence suggests that circulating branched-chain amino acids (BCAAs) are associated with T2D. While excess BCAAs are shown to be harmful to neurons, its connection to AD is poorly understood. Here we show that individuals with AD have elevated circulating BCAAs and their metabolites compared to healthy individuals, and that a BCAA metabolite is correlated with the severity of dementia. APP_Swe_ mouse model of AD also displayed higher plasma BCAAs compared to controls. In pursuit of understanding a potential causality, BCAA supplementation to HT-22 neurons was found to reduce genes critical for neuronal health while increasing phosphorylated Tau. Moreover, restricting BCAAs from diet delayed cognitive decline and lowered AD-related pathology in the cortex and hippocampus in APP/PS1 mice. BCAA restriction for two months was sufficient to correct glycemic control and increased/restored dopamine that were severely reduced in APP/PS1 controls. Treating 5xFAD mice that show early brain pathology with a BCAA-lowering compound recapitulated the beneficial effects of BCAA restriction on brain pathology and neurotransmitters including norepinephrine and serotonin. Collectively, this study reveals a positive association between circulating BCAAs and AD. Our findings suggest that BCAAs impair neuronal functions whereas BCAA-lowering alleviates AD-related pathology and cognitive decline, thus establishing a potential causal link between BCAAs and AD progression.

## 1. Introduction

Alzheimer’s disease (AD) is an irreversible neurodegenerative disorder ranking as the sixth leading cause of mortality in the US affecting over 5 million Americans [1]. Unfortunately, no cure or effective treatments currently exist that can slow down the progression of AD. It is well established that Type 2 diabetes (T2D) is a strong risk factor for AD development [2]. Since these diseases share a number of key abnormal features such as insulin resistance, hyperglycemia, inflammation, and vascular dysfunction [3], it is reasonable to speculate that any perturbed neural and/or nutrient and related hormonal regulations observed in T2D may also underlie the pathophysiological characteristics found in AD.

Branched-chain amino acids (BCAAs; leucine, isoleucine, valine) are essential amino acids we need to obtain from our diet. They are elevated in obesity and T2D independent of protein intake [4,5,6,7], and are found to be the earliest predictive marker for future risk of T2D [8]. More recent findings in both human and animal studies suggest that BCAAs can impair insulin sensitivity and glycemic control [9,10,11,12,13,14,15]. Interestingly, current literature supports an important role of BCAAs in brain functions as well. Supplementation with BCAAs or their derived metabolites can induce neural oxidative stress [16,17] and mitochondrial dysfunction [18,19]. In support of these findings, individuals with maple syrup urine disease (MSUD), a rare genetic disorder that causes a mutation in genes encoding branched-chain α keto-acid dehydrogenase (BCKDH) complex, the rate-limiting enzyme in BCAA degradation pathway, have nearly 10-fold higher BCAAs and their toxic keto-acids in plasma compared to healthy controls [20,21], leading to serious neurological impairments.

Further evidence correlating BCAAs to brain function comes from the observation that the enzyme branched-chain aminotransferase (BCAT) converts BCAAs to glutamate, an excitatory neurotransmitter [22]. Moreover, BCAAs compete for the same large amino acid transporter (LAT) with aromatic amino acids like phenylalanine, tyrosine, and tryptophan—the precursors of neurotransmitters like dopamine, norepinephrine, and serotonin [23]. Thus, excess circulating BCAAs may lead to production of too much glutamate in the brain that can contribute to excitotoxicity, as well as trigger a significant imbalance and reduction of some key neurotransmitters. Lastly, hyperactivation of mTOR signaling is linked to AD and has been shown to impair insulin signaling both in the periphery and the brain [6,24,25,26]. This is supported by a recent study from Caccamo and colleagues [27] that showed Tg2576 mice lacking one copy of mTOR restored brain insulin signaling that was accompanied by improved memory and decreased Aβ and Tau proteins. It is important to emphasize that leucine is a potent stimulator of mTOR [28]. Interestingly, all of these neuronal and cellular defects that can be triggered by BCAAs represent the main pathological features of AD. This lends further support to the concept that defective BCAA control seen in T2D may also exist in AD, however our current knowledge on the role of BCAAs and their metabolism in the onset of AD is poorly understood. In addition, a limited number of studies reveal conflicting reports on the association between BCAAs and AD [29,30,31,32,33,34,35,36,37,38,39,40], making it imperative to investigate this relationship further.

In the present study, we sought to determine the role of BCAAs in the pathogenesis of AD. To this end, serum samples from older individuals with or without AD were obtained through Texas Alzheimer’s Disease Research Care Consortium (TARCC). Samples from patients with T2D alone or T2D in conjunction with AD were also acquired to assess BCAA-related metabolic signatures and examine the relative changes between different disease conditions. BCAA metabolism was further studied in a widely used APP_Swe_ transgenic mouse model to determine plasma BCAA levels and their degradation efficiency in liver, an organ with highly active BCAA breakdown as previously shown by our group and others [41,42]. To better understand the role of BCAAs and begin to establish a potential mechanistic link to AD-related pathology and cognitive deficits, HT-22 hippocampal neuronal cell line was used to investigate the effects of BCAA supplementation on the regulation of genes commonly found to be impaired in AD. Lastly, in vivo experiments with two widely used transgenic mouse models of AD—APP/PS1 and 5xFAD—were conducted to test whether a dietary or pharmacological approach that lowers plasma BCAAs can alleviate AD progression.

## 2. Materials and Methods

### 2.1. Human Data Collection

Serum samples from either healthy males or males with AD, T2D, or T2D+AD (age ≥ 60 years old; *n* = 10/group) were obtained from the Texas Alzheimer’s Disease Research Care and Consortium (TARCC) [43,44]. Scores for CDR and Mini-Mental State Examination (MMSE) for the participants were obtained from TARCC.

### 2.2. Animals

Three popular transgenic mouse models of AD were used in this study: APP_Swe_ mice that overexpress a mutant form of APP with the Swedish mutation (*Tg2576*; Charles River Laboratories); APP/PS1 mice that overexpress chimeric mouse/human APP and a mutant human presenilin 1 (*B6*;*C3-Tg(APP_Swe_*,*PSEN1dE9)85Dbo/Mmjax*; The Jackson Laboratory; Stock # 34829); 5xFAD mice that overexpress human APP with Swedish (K670N, M671L), Florida (I716V), and London (V717l) mutations and human presenilin 1 with two mutations (*B6SJL-Tg(APPSwFlLon*,PSEN1*M146L*L286V)6799Vas/Mmjax; The Jackson Laboratory; Stock # 34840). APP_Swe_ and littermate W T mice were group-housed while APP/PS1, 5xFAD, and the corresponding littermate WT mice were single-housed due to their aggressive nature. All animals except APP/PS1 and WT mice were provided ad libitum with a regular chow diet (22% protein, 63% carbohydrate, and 15% fat; Cat # 5V5R; LabDiet) and water unless otherwise stated. All mice were housed in the animal facility room with a temperature of 23 ± 2 °C and 12:12 h light/dark cycle. All studies were conducted in accordance with the National Institutes of Health’s Guide for the Care and Use of Laboratory Animals, and the protocol was approved by the Institutional Animal Care and Use Committee (IACUC) at Texas Tech University.

### 2.3. Validation of BCAA Metabolism in AD Mouse Model

8-month-old male APP_Swe_ (*n* = 13) and WT mice (*n* = 7) were euthanized after overnight fasting and blood was collected in EDTA-coated tubes via cardiac puncture, and plasma was separated by centrifugation to detect BCAA levels. Liver and other tissues were harvested, snap-frozen in liquid nitrogen, and stored at −80 °C to examine BCAA catabolism as described below.

### 2.4. BCAA Restriction Experiment

11-month-old APP/PS1 and WT mice were first subjected to Y-maze to assess their cognitive function at baseline and to blood collection from tail nick for BCAA measurement. Basal blood glucose was also monitored by a hand-held glucometer (AlphaTRAK2) following 4 h fast. Then, the animals were placed on either a purified control diet or a control diet with BCAA restriction for two months (WT control, *n* = 10 (5 M, 5 F); WT BCAA restriction, *n* = 10 (5 M, 5 F); APP/PS1 control, *n* = 8 (5 M, 3 F); APP/PS1 BCAA restriction, *n* = 8 (5 M, 3 F)). For BCAA restriction, 50% of the BCAAs (leucine, isoleucine, valine) was removed from the control diet. The limited amount of BCAAs and treatment duration are based on a recent study [4,5]. The custom diet with restricted BCAAs (Research Diet) was iso-caloric and iso-nitrogenous to the control diet. Both diets were made from purified sources with accurately measured ingredients from the vendor, and details on the nutritional composition of both diets are shown in Table 1. Weekly food intake and body weight (twice per week) were monitored to observe any changes in energy balance that may be brought about by limited BCAA intake. At the end of two months, we performed Y-maze again with these mice to detect any post-treatment effects on cognitive function. A couple days later, they were sacrificed after 4 h fasting by isoflurane overdose and cervical dislocation. Trunk blood collected at sacrifice was not usable due to heavy hemolysis. Brain was harvested fresh, and the neocortex and hippocampus were separated quickly in an ice-cold PBS solution. Micropunches were taken from both brain regions for monoamine analysis, and the rest of the cortical and hippocampal tissues were kept frozen in −80 °C for protein and gene analyses. Liver and other tissues were harvested, snap-frozen in liquid nitrogen, and stored at −80 °C to examine BCAA catabolism.

### 2.5. BT2 Experiment

6–8-week-old WTs or 5xFAD male mice were separated into four groups: WT vehicle (*n* = 6), WT BT2 (*n* = 6), 5xFAD vehicle (*n* = 4), and 5xFAD BT2 (*n* = 4). After checking blood glucose and collecting tail blood at baseline after 2.5 h fasting, animals were injected with BT2 (40 mg/kg ip; Millipore Sigma, Cat # 34576-94-8), a BCAA-lowering compound, daily in the morning for 30 consecutive days. The dose is based on what has been used in previous studies that demonstrated an effective lowering of plasma BCAAs in rodents in the context of normal, insulin resistance/diabetes, or heart failure models [12,15,45,46,47]. Treatment duration ranges from 1 week to 7–8 weeks depending on studies, so we speculated that 30 days treatment would be safe and likely be able to show positive effects if BCAA-lowering is indeed a critical mechanism in delaying AD pathophysiology. BT2 was dissolved in DMSO, then diluted to a final concentration of 5% DMSO, 10% Cremophor EL, and 85% 0.1 M sodium bicarbonate, at pH 9 for delivery. Daily body weight and weekly food intake were monitored during the treatment. At the end of 30 days, mice were euthanized using isoflurane overdose followed by cervical dislocation. Brain was harvested fresh, and the neocortex and hippocampus were separated quickly in an ice-cold PBS solution. Micropunches were taken from both brain regions for monoamine analysis, and the rest of the cortical and hippocampal tissues were kept frozen in −80 °C for protein and gene analyses. Blood was also collected at sacrifice for BCAA measurements.

### 2.6. HT-22 In Vitro Experiment

The immortalized mouse hippocampal neuronal HT-22 cells were purchased from Millipore Sigma, Burlington, MA, USA, Cat # SCC129. These neuronal precursors were maintained in Dulbecco’s Modified Eagle’s Medium (DMEM; Thermo Fisher Scientific, Waltham, MA, USA, Cat # 11995073) supplemented with 10% fetal bovine serum (Sigma-Aldrich, Inc., St. louis, MO, USA Cat # SH30071.03), 1% Penicillin-Streptomycin Solution (Sigma, St. Louis, USA, Cat # A5955) at 37 °C and 5% (*v*/*v*) CO_2_. Before BCAA supplementation, 80% confluent HT-22 cells were differentiated in B-27™ Plus Neuronal Culture System media (Thermo Fisher Scientific, Waltham, MA, USA, Cat # A3653401) for 24 h. All treatments were performed in differentiation media. To test the role of BCAAs on mature neurons, differentiated HT-22 neurons were supplemented with 0, 1, 5, or 10 mM of BCAAs (Millipore Sigma, Burlington, MA, USA; L-Leucine, Cat # 61819; L-Isoleucine, Cat # 58879; L-Valine, Cat # 94619) for 24 h. These doses were selected based on our pilot data on AD mice and others’ findings [48,49,50] that BCAA levels in the cerebrospinal fluid (CSF) are lower by about 10–20 fold compared to those found in plasma, thereby allowing us to estimate that a concentration between 5 and 10 mM in the culture media would lead to 500–900 µM of BCAAs in neuronal cells, similar to the levels found in AD mouse brain. Following treatment, HT-22 neurons were harvested for gene expression analysis by RT-qPCR. Genes involved in mitophagy, autophagy, mitochondrial biogenesis, synapse formation, mitochondrial dynamics, and retromer trafficking were determined. Protein was also extracted to probe GSK3β and Tau proteins. In a separate cohort, we sought to assess potential detrimental effects of BCAA supplementation on glucose metabolism as this is a reliable readout for neuronal health. To this end, 10 mM of BCAAs was supplemented since this was found to be the most effective dose of BCAAs in impairing the neuronal functions as described above. HT-22 cells were differentiated into mature neurons and were treated with 0 or 10 mM of BCAAs for 24 h. Another group of HT-22 neurons was treated with 25 mM glucose, an established neurotoxicity in vitro model, for 24 h to compare the effects of BCAA supplementation. Then, cells were harvested and processed for analysis of genes relevant to glycolytic pathway and inflammation.

### 2.7. Y-Maze Behavioral Test

Y-maze comprised three arms at 120° angles. The arms were designated as ‘A’, ‘B’, and ‘C’. Before starting a testing session, a data acquisition system (ANY-maze video tracking system) was set up to properly track a mouse in the maze. At a time, a single mouse was placed into one of the arms in the Y-maze facing the center. The mouse was allowed to explore the maze freely for 2 min. Once the session was complete, the mouse was gently returned into its home cage. The Y-maze was cleaned thoroughly between each trial with 70% ethanol and water. Once all mice had completed the exploration of the Y-maze, spontaneous alternation (SA) was calculated with the following formula:

% SA = # SA/(total # of arm entries − 2) × 100(1)

### 2.8. Quantification of Brain Monoamines

Micropunched samples from the cortex and hippocampus were homogenized in 200 µL of 0.05 M perchloric acid using a Kontes electrical homogenizer (Kontes, Vineland, NJ, USA). An aliquot of the homogenate was saved for the BCA protein assay (Pierce Biotechnology, Rockford, IL, USA). The remaining homogenate was centrifuged at 18,000× *g* for 7 min. Then, 15 µL of the supernatant was injected into the High Performance Liquid Chromatography system with 0.05 M DHBA as the internal standard. We used a Shimadzu Prominence UFLC system (Shimadzu, Columbia, MD, USA), a C-18 reverse phase 5 µm ODS column (Phenomenex, Torrance, CA, USA) and a dual glassy carbon electrode connected to a BAS LC-6C detector (BASi, West Lafayette, IN, USA). A RE-6 Ag/AgCl reference electrode set at 0.65 V was used and the range of the detector was 1 nA. Chromatograms were analyzed using Class-VP software Version 7.4-SP3. Concentration of monoamine neurotransmitters and their metabolites (i.e., Norepinephrine, NE; Dopamine, DA; DOPAC; Serotonin, 5-HT; 5-HIAA) was normalized by the protein concentration of a sample and was expressed as pg/µg protein.

### 2.9. Untargeted Metabolomics

Human serum samples were prepared by the following procedures. First, methanol as an organic solvent was mixed with serum, then, chloroform was added. To separate the aqueous fraction from organic phase, water was added and followed by centrifugation. Afterwards, the aqueous phase (top layer) was separated for metabolite analysis. Next, the liquid chromatography-mass spectrometry (LC-MS/MS) analysis was performed using ACQUITY UPLC HSS T3 column (2.1 mm × 100 mm, 1.7 µm, 130 A) with a 25 min gradient for metabolites analysis. The MS analysis was performed using Q-Exactive HF mass spectrometer (Thermo Fisher Scientific, Waltham, MA, USA) in both positive and negative ion modes with the full-scan MS (*m*/*z* 50-7500) and MS/MS by HCD using stepped collision energy of 20–60%. The data analysis for identification and quantification of metabolites including BCAAs, their keto acids, and oxidized metabolites was done by Compound Discoverer 3.1 software.

### 2.10. BCAA Assay

To measure plasma BCAA levels in AD or WT mice, a spectrophotometric assay that measures NADH generated from BCAA oxidation was used as previously described [51]. Briefly, 7 leucine standards were prepared by adding leucine into ddH_2_O at a concentration of 100, 200, 400, 600, 800, 1000, and 2000 µM. For 10 mL of reaction buffer, 1 mL of 0.1 M glycine (pH 10.5), 1 mL of 0.1 M KCl (pH 10.5), and 1 mL of 0.1 M KOH (pH 10.5) were added to 7 mL of ddH_2_O. Additionally, for every 10 mL of buffer, 0.1 mL of 0.2 M EDTA (pH 8) was added. The above calculation was scaled up or down depending on the number of samples given that 270 µL of buffer was added to each well of a 96 well plate. Finally, the pH of the solution was kept between 10.5 and 10.7. NAD solution was made by adding 20 mg of NAD in 251 µL of sodium carbonate (pH 10.7). 10 µL of the sample or standard was added, followed by 10 µL of freshly prepared NAD to 270 µL of buffer except for the blank. Background absorbance was taken at 340 nm afterwards. After adding 10 µL of leucine dehydrogenase (200 U/mL) to each well, the 96-well plate was incubated in the dark for 30 min and reaction absorbance was taken at 340 nm. After subtracting the values of background from those of reaction, a standard curve was made, and BCAA concentrations from samples were calculated accordingly and expressed in μM.

### 2.11. Western Blots

Harvested HT-22 cells, brain, or liver tissues were homogenized in radioimmune precipitation assay (RIPA) buffer (Cell Signaling, Danvers, MA, USA, Cat # 9806) with a protease inhibitor. Protein from the homogenized mixture was extracted after a two-step centrifugation process (5 min at 5000 rpm and 15 min at 13,000 rpm) and measured by bicinchoninic acid (BCA) protein assay (Thermo Fisher Scientific, Waltham, MA, USA, Cat # 23225). 30 μg protein extracts were separated using sodium dodecyl sulfate-polyacrylamide gel electrophoresis (SDS-PAGE), transferred on to a polyvinylidene difluoride (PVDF) membrane, blocked by 5% non-fat milk in TBST for an hour followed by overnight incubation with a primary antibody for BCATm (Abcam, Waltham, MA, USA, Cat # ab95976), BCKDH (Abcam, Cat # ab138460), BCKDH kinase (Abcam, Cat # ab128935), pGSK-3α/β (Ser21/9) (Cell Signaling, Danvers, MA, USA, Cat # 9331S), GSK-3α/β (Cell Signaling, Cat # 5676S), pTau S202 (Abcam, Cat # ab108387), pTau S396 (Abcam, Cat # ab109390), pTau T205 (Thermo Fisher Scientific, Waltham, MA, USA, Cat # 44-738G), Tau (Cell Signaling, Cat # 30328), Aβ-42 (Cell Signaling, Cat # 8243S), PSD95 (Cell Signaling, Cat # 2507S), IDE (Invitrogen, Waltham, MA, USA, Cat # PA5-29349), TH (Cell Signaling, Cat # 58844) or GAPDH (Cell Signaling, Cat # 2118S) at 1:1000 dilution. To visualize protein bands, the membrane was immunoblotted with appropriate secondary antibody conjugated with horseradish peroxidase (1:4000 dilution) and treated with clarity western ECL substrate reagent (Bio-Rad, Hercules, CA, USA, Cat # 170-5061). ImageJ software was used for the quantification of protein bands.

### 2.12. ELISA

A commercially available ELISA kit (ThermoFisher Scientific, Waltham, MA, USA, Cat # KHB3442) was used to measure recombinant human Aβ-42 in the neocortex of WT and APP/PS1 mice in BCAA restriction experiment. Per the manufacturer’s instructions, proteins from cortical tissues were first extracted with the lysis buffer solution containing guanidine hydrochloride before running the assay for detection of human Aβ-42. The sensitivity of the assay was 15.6 pg/mL. Cortical human Aβ-42 could not be measured in BT2 experiment because the whole neocortex tissues were already used for Western blots.

### 2.13. Real-Time Quantitative PCR (RT-qPCR)

Total RNA was extracted either from the cells or tissues using the RNeasy^®^ Plus Universal Mini Kit (Qiagen, Germantown, MD, USA, Cat # 73404). cDNA was synthesized from 1 μg total RNA using the iScript^TM^ Reverse Transcription Supermix (Biorad, Cat # 1708841). cDNA was diluted 1:20 with nuclease-free water and stored at −20 °C. cDNA, at a final concentration of 5 ng/µL, was amplified using SsoAdvanced™ Universal SYBR^®^ Green Supermix (Bio-Rad, Cat # 172-5271). The RT-qPCR reaction mix had a final volume of 20 µL: 25 ng of cDNA, 450 nM of the forward and reverse primers, and 10 µL of 1X SYBR Green. Real-time qPCR was performed in a Bio-Rad CFX RT-PCR detection system. The relative mRNA expression was determined using the ΔΔCt method, while GAPDH or beta-2 microglobulin (B2M) gene was used as a reference gene. Primers used in this study are listed in Table 2.

### 2.14. Statistical Analysis

Human serum BCAAs and their metabolites, plasma BCAAs and hepatic enzymes in APP_Swe_ and WT mice, and plasma BCAAs from BCAA restriction experiment were analyzed by student’s *t*-test. Correlation test was performed for the associations between BCAA-derived metabolites and CDR scores or MMSE scores, and Pearson correlation coefficient was calculated. Blood glucose, plasma and cortical BCAAs and Y-maze results in BCAA restriction experiment, proteins and mRNAs in liver, hippocampus, or cortex in both BCAA restriction and BT2 experiments, and mRNAs in HT-22 cells were analyzed by two-way ANOVA to evaluate the effect of treatment, genotype, and interaction, followed by Tukey’s post hoc test. For monoamines in the hippocampus and cortex, two comparisons were made (WT veh vs. 5xFAD veh; 5xFAD veh vs. 5xFAD BT2) for which Bonferroni-adjusted multiple comparison tests were performed with *p* value set at less than 0.025. Body weight and food intake in both BCAA restriction and BT2 experiments, and blood glucose in BT2 experiment were analyzed by repeated measures ANOVA followed by Bonferroni post hoc test. Data are expressed as Mean ± SEM. Significant difference between groups was set at *p* < 0.05. Statistical software GraphPad Prism 9 was used to analyze the data.

## 3. Results

### 3.1. Serum BCAAs and Their Metabolites Are Associated with AD

**Figure 1:**
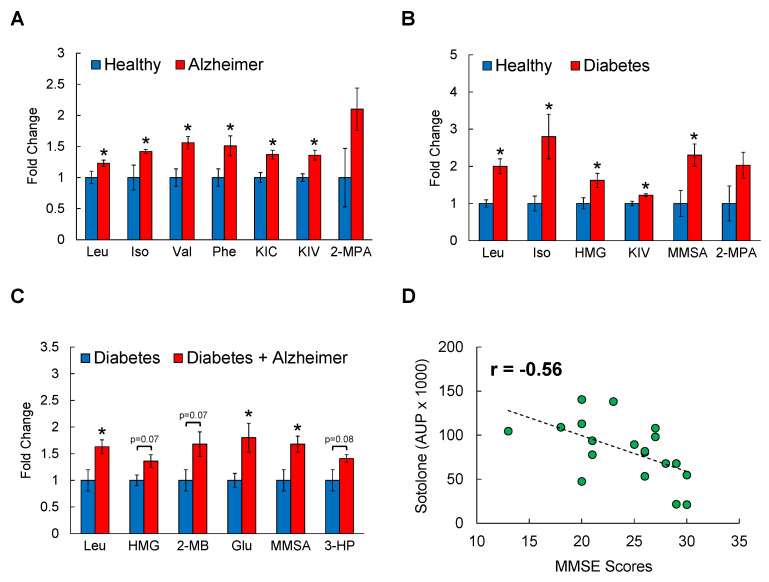
Circulating BCAA levels and their metabolites are elevated in individuals with AD. Serum samples from age- and BMI-matched healthy controls, patients with AD, T2D, or with T2D+AD after overnight fasting were analyzed by LC/MS. (**A**) Healthy vs. AD; (**B**) Healthy vs. Diabetes; (**C**) Diabetes vs. Diabetes+AD. Data shown are fold changes normalized to the mean of healthy or Diabetes controls; *n* = 10 males/group; mean age = 72 ± 3 years old; * *p* < 0.05. (**D**) Correlation analysis between serum sotolone, a metabolite of isoleucine, and MMSE scores. Iso: Isoleucine; HMG: 3-methylglutaryl (metabolite of leucine); KIV: ketoisovalerate (metabolite of valine); KIC: ketoisocaproate (metabolite of leucine); MMSA: Methyl-melonatesemialdehyde (metabolite of valine); Phe: Phenylalanine; 2-MPA: 2-methylpropanoic acid (metabolite of valine); 3-HP: 3-OH-Propanoate (metabolite of valine); MMSE: Mini-Mental State Examination.

To first determine a possible link between circulating BCAA levels and AD, serum samples from male individuals with AD or BMI- and age-matched healthy individuals were analyzed through untargeted metabolomics by LC-MS (Supplementary Table S1). From 95 metabolites that are found to be differentially regulated, Compound Discoverer database identified BCAA metabolism as one of the most affected biological pathways in AD patients. As shown in Figure 1A, all BCAAs including leucine, isoleucine, and valine as well as their derivatives such as keto-isocaproate (KIC; metabolite of leucine) and keto-isovalerate (KIV; metabolite of valine) were significantly higher in AD group compared to those in healthy controls, with an increase ranging from 20–50%. Importantly, other amino acids in the serum except for phenylalanine were not different between healthy and AD individuals (Supplementary Table S2). T2D is known to share several abnormal features with AD, and it is now widely accepted that systemic BCAA levels are elevated in insulin-resistant or diabetic state [4,5,6,7,8,52]. To test if having both conditions magnify BCAA-raising effects, samples from patients with T2D or T2D+AD were also obtained and subjected to metabolomics platform. Serum BCAAs (100–180%) and their oxidized intermediates including 3-methylglutarylcarnitine (HMG; metabolite of leucine; 60%) and methylmelonate semialdehyde (MMSA; metabolite of valine; 120%) were significantly elevated in T2D group compared to those in healthy control group as expected (Figure 1B). Interestingly, individuals with both T2D and AD displayed even higher levels of leucine (60%) and its metabolite HMG (35%), glutamate (80%), and oxidized metabolites of valine such as 2-methylbutyryl (2-MB; 65%), MMSA (60%), and 3-hydroxypropanoate (3-HP; 40%) compared to individuals with T2D alone (Figure 1C), suggesting an association between BCAAs and AD that extends beyond the effect of T2D. To examine this relationship further, we obtained Clinical Dementia Rating (CDR) and Mini-Mental State Examination (MMSE) scores from the same healthy and AD individuals and conducted a correlation analysis between the scores and the serum levels of BCAA-related metabolites. The sum of CDR scores ranges from 0 (normal) to 18 (severe dementia), and in MMSE a score between 25–30 points to normal cognition and a score less than 12 indicates severe dementia. While the results from either test do not strictly confirm the presence or development of AD, they do reflect the neuropsychological symptoms and the severity of dementia that can be found in neurodegenerative disorders like AD. While 2-methylbutyrylcarnitine (2-MBC), a metabolite of isoleucine, showed a non-significant association with both CDR scores (r = 0.53) and MMSE scores (r = −0.46; not shown), another metabolite of isoleucine, sotolone, was found to be significantly, and inversely correlated with MMSE scores (r = −0.56, *p* < 0.05; Figure 1D). Collectively, these findings strongly suggest that circulating BCAAs and/or their metabolites are positively associated with AD progression and/or related cognitive impairment in humans.

### 3.2. BCAA Catabolism Is Impaired in APP_Swe_ Transgenic Mice

**Figure 2:**
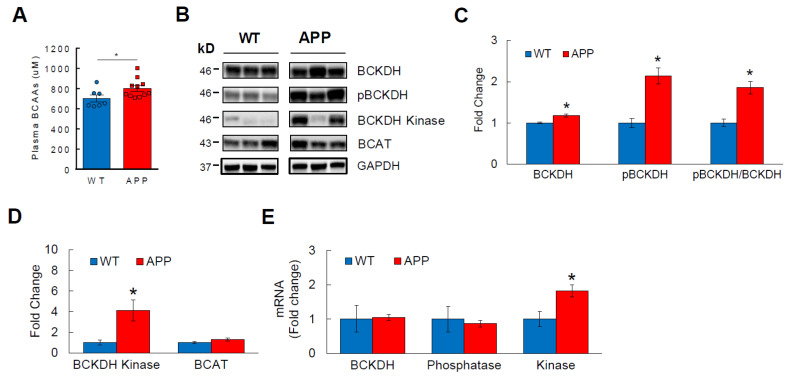
BCAA metabolism is impaired in transgenic APP_Swe_ mice. 8-month-old WT or APP_Swe_ AD mice were sacrificed after overnight fasting. (**A**) Fasting plasma BCAA levels measured by spectrophotometric assay. (**B**) Western blots showing liver protein abundance of BCKDH, the rate-limiting enzyme in BCAA breakdown; phosphorylated, inactive state of BCKDH (pBCKDH); BCKDH kinase, an enzyme that phosphorylates BCKDH; and BCAT, an enzyme involved in the reversible first step in BCAA degradation. (**C**) Analysis of BCKDH, pBCKDH, and the inactivity index (pBCKDH/BCKDH ratio). (**D**) Analysis of BCKDH kinase and BCAT in liver. (**E**) mRNA expression analysis of BCKDH, BCKDH phosphatase, and BCKDH kinase via RT-qPCR in liver normalized to GAPDH. *n* = 7–13 males/group; Analyzed by student’s *t*-test. * *p* < 0.05. Phosphatase—BCKDH phosphatase; Kinase—BCKDH kinase.

Transgenic familial AD mice share remarkable similarities with humans in regard to the formation of amyloid plaques and/or neurofibrillary tangles and related cognitive decline, thus serving as a convenient tool to study the disease pathophysiology [53]. APP_Swe_ mouse (also known as Tg2576) is one of the most widely used mouse models of AD that overexpresses human amyloid precursor protein (APP) containing Swedish mutation and shows a cognitive impairment as early as 6 months of age [54]. We measured plasma BCAA levels from 8-month-old APP_Swe_ mice to determine if a relationship similar to what we observed in humans exists in these mice. Indeed, plasma BCAAs were significantly elevated in APP_Swe_ mice compared to WT controls (Figure 2A), establishing the basis to use transgenic AD mice such as this model to further investigate the disease progression and its underlying mechanisms. We and others have previously shown that circulating BCAA levels are regulated primarily by hepatic BCAA catabolism [41,42]. Branched-chain α-ketoacid dehydrogenase (BCKDH) is the rate-limiting enzyme in BCAA breakdown that becomes inactive when phosphorylated by BCKDH kinase [41]. Protein analysis from liver demonstrated significantly higher pBCKDH in APP_Swe_ mice compared to that in WTs, and so was the inactivity index as expressed by pBCKDH/BCKDH ratio (Figure 2B,C). In support of these results, APP_Swe_ mice also showed higher protein expression of BCKDH kinase without affecting the first enzyme in BCAA degradation pathway, branched-chain aminotransferase (BCAT; Figure 2D). This is further confirmed by a markedly increased mRNA of hepatic BCKDH kinase in APP_Swe_ mice (Figure 2E). Altogether, these data suggest that the association between high circulating BCAA levels and AD extends to a transgenic mouse model such as APP_Swe_, and that this may be primarily due to impaired hepatic BCAA catabolism in AD.

### 3.3. BCAA Supplementation Induces AD-Like Changes and Disrupts Cellular Functions in HT-22 Neurons

**Figure 3:**
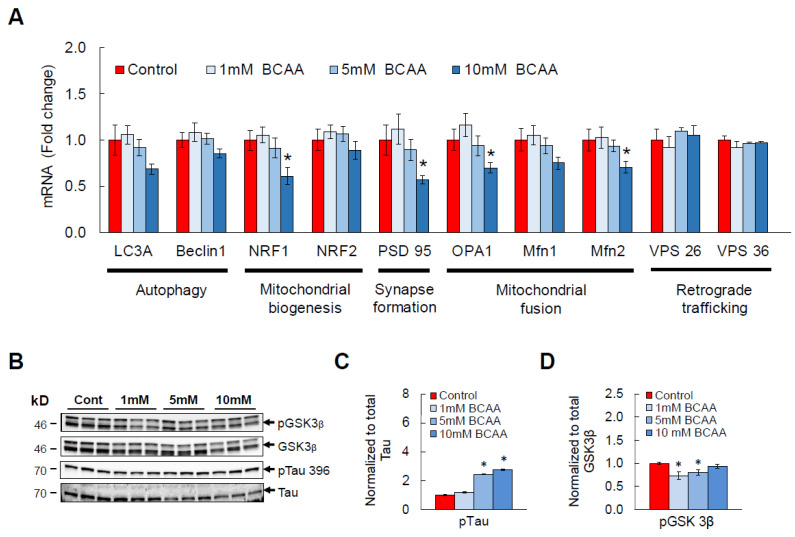

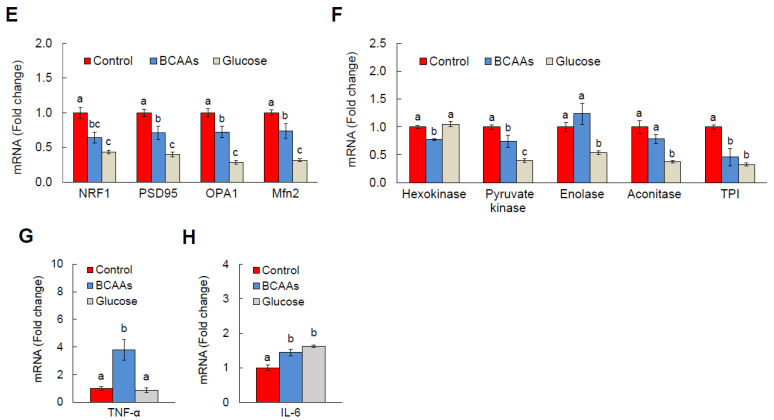
BCAA treatment induces AD-related changes in vitro. Differentiated HT-22 hippocampal neurons were supplemented with a mixture of BCAAs in the culture media for 24 h. (**A**) mRNA expression of genes involved in autophagy, mitochondrial biogenesis and fusion, synapse formation, and retrograde trafficking. (**B**) Western blots showing pGSK3β, total GSK3β, pTau 396, total Tau. (**C**) Analysis of Tau phosphorylation at residue 396 and (**D**) pGSK3β. (**E**–**H**) In a separate cohort, differentiated HT-22 neurons were exposed to either vehicle or 10 mM BCAAs in the media for 24 h. Another group was exposed to 25 mM glucose to induce glucotoxicity, a widely used neurotoxicity model, as a comparison. (**E**) mRNA levels for neuronal health genes as described above were analyzed. (**F**) Genes critical for glycolytic pathway. (**G**) mRNAs of TNF-α and (**H**) IL-6. All mRNAs were normalized to B2M. *n* = 6/group; * *p* < 0.05 compared to Control. Groups with different letters (i.e., a, b, c) are significantly different from each other with *p* < 0.05.

Elevated systemic levels of BCAAs and their metabolites in AD raise a possibility that BCAAs and AD are causally linked. To test this, 0, 1, 5, or 10 mM of a mixture of BCAAs (leucine, isoleucine, valine) was supplemented to differentiated HT-22 hippocampal neurons for 24 h. Middle and high doses were used to target BCAA concentrations found in an AD mouse brain (See Section 2). Our in vitro results showed that BCAA exposure dose-dependently downregulates mRNA of neuronal health markers such as LC3A (autophagy), NRF1 (mitochondrial biogenesis), PSD 95 (synapse formation), as well as OPA1, Mfn1, and Mfn2 (mitochondrial fusion; Figure 3A). It is important to note that these genes are among the ones commonly found to be impaired in AD brain, and these impaired neuronal functions in AD are clearly demonstrated by increased oxidative stress and a dramatically reduced synaptogenesis and ATP production [55,56]. Moreover, we observed a significant increase in pTau in BCAA-treated HT-22 neurons compared to that in vehicle-treated neurons (Figure 3B,C), and this is most likely attributed to a significant decrease in phosphorylated, inactive state of GSK3β (Figure 3D), an enzyme responsible for phosphorylating Tau protein that leads to formation of paired helical filaments (PHF) and neurofibrillary tangles (NFT). Neurons have a high demand for glucose for functions including synthesis of ATP and neurotransmitters [57], thus making proper glucose metabolism crucial for the overall health and communication between neurons. In line with this, glucose metabolic defect has been often observed in AD brain and neurons [58,59]. To further understand the detrimental effects of BCAA exposure on neuronal function and establish a mechanistic link to AD, we treated differentiated HT-22 neurons with BCAAs and examined the glycolysis pathway. A significant downregulation of genes responsible for mitochondrial biogenesis and fusion as well as synapse formation was verified in an independent cohort (Figure 3E). As expected, BCAA supplementation for 24 h was enough to dramatically lower mRNAs that encode enzymes involved in glycolysis, including the rate-limiting enzymes hexokinase and pyruvate kinase (Figure 3F). Interestingly, the harmful effects of BCAAs followed those from a high-glucose treatment, a widely used in vitro neurotoxicity model for establishing neuronal stress, disrupted cellular metabolism, and apoptosis [60,61,62]. In agreement with BCAA-induced neuronal dysfunction, mRNA abundance of pro-inflammatory mediators TNF-α and IL-6 was markedly increased compared to vehicle-treated HT-22 neurons (Figure 3G,H). These findings support the concept that BCAAs may cause multiple neuronal dysfunctions as commonly observed in AD at least partly by impairing cellular glycolytic and bioenergetic pathways in neurons.

### 3.4. BCAA-Restriction Diet Delays Onset of Cognitive Decline in APP/PS1 Mice

**Figure 4:**
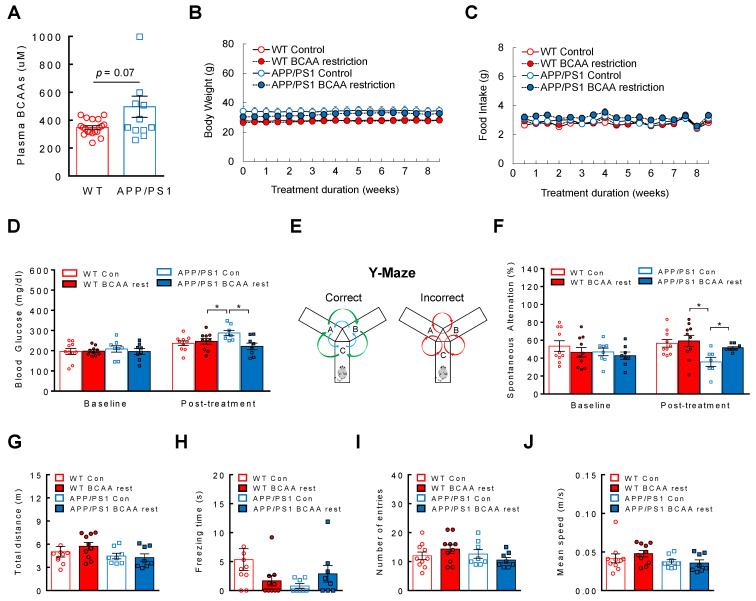
BCAA restriction delays the onset of cognitive deficit in APP/PS1 mice. 11-month-old WT or APP/PS1 mice without cognitive impairment were placed on a control diet or BCAA-restricted (50%) diet, iso-caloric and iso-nitrogenous, for two months. (**A**) Plasma BCAA levels at baseline. (**B**) BCAA restriction does not affect body weight or (**C**) Food intake. (**D**) Blood glucose before and after treatment. (**E**) Schematic of Y-maze behavioral test. (**F**) Spontaneous alternation (%) before and after treatment. (**G**) Total distance traveled, (**H**) Freezing time, (**I**) Number of arm entries, and (**J**) Mean speed during Y-maze test. WT control or BCAA restriction group (*n* = 10/group); APP/PS1 control or BCAA restriction group (*n* = 8/group). * *p* < 0.05.

Given the dose-dependent downregulation of neuronal health markers and upregulation of pTau by BCAA supplementation, it is possible that BCAAs are causally linked to AD development. Considering already elevated plasma BCAA levels in AD, we determined that limiting BCAA consumption rather than providing more BCAAs would be a more physiological approach to test the hypothesis. To this end, WTs and APP/PS1 transgenic mice were placed on either a control diet or a customized diet that is deficient of individual BCAAs by 50% for two months. The special diet was formulated to restrict the amount of all three BCAAs while being iso-caloric and iso-nitrogenous compared to the control diet (Table 1). We predicted that if BCAAs are indeed a significant contributor to AD pathogenesis, then lowering BCAA intake would at least partly slow down the disease progression. APP/PS1 mice displayed a tendency of higher plasma BCAA levels compared to WTs before dietary treatment (Figure 4A), thus confirming our earlier data from APP_swe_ mouse model. BCAA levels from the mouse cortex were not different between groups (Supplementary Figure S1) which is in agreement with no change in BCAAs in cortical homogenates from 3xTg AD mice placed on a normal chow or BCAA restriction diet [63]. Diet change did not affect body weight or food intake for the entire treatment duration (Figure 4B,C). Metabolic dysregulation such as insulin resistance or impaired glycemic control is often associated with AD [64,65]. While there was no difference in blood glucose between groups at baseline, after two months we observed a significantly increased blood glucose in control diet-fed APP/PS1 mice compared to that in WT controls, as well as their own baseline (main effect of treatment: F(1,32)= 4.24, *p* = 0.047; interaction: F(1,32) = 7.59, *p* = 0.0096; Figure 4D). Interestingly, BCAA restriction was able to prevent the rise of blood glucose in APP/PS1 mice (Figure 4D). Next, we determined the effects of limiting BCAA intake on the cognitive function in these mice. Y-maze (Figure 4E) is a widely used behavioral test to assess working memory. APP/PS1 mice did not show any differences in spontaneous alternation compared to WTs at baseline, most likely because they were not old enough to show any cognitive dysfunction (ex. spatial learning) which usually occurs between 12–13 month of age (Figure 4F). As expected, they displayed poor memory after two months on a control diet as evidenced by lower spontaneous alternation, but APP/PS1 mice on BCAA-restricted diet resisted the decline and they were not different from WT mice on either diet (main effect of genotype: F(1,31) = 8.39, *p* = 0.007; Figure 4F). To rule out the possibility that the behavioral outcome induced by BCAA restriction is partly due to changes in the mobility or distraction of the animals, we carefully examined the total distance (Figure 4G), freezing time (Figure 4H), number of entries (Figure 4I), and mean speed (Figure 4J), and none of the parameters were found to be markedly different between groups. These results suggest that restricting BCAA consumption substantially delays AD progression independent of changes in energy balance, and this cognitive benefit is associated with improved glycemic control.

### 3.5. BCAA-Restriction Diet Lowers AD-Related Pathology and Restores Neurotransmitter Levels in the Cortex and Hippocampus in APP/PS1 Mice

**Figure 5:**
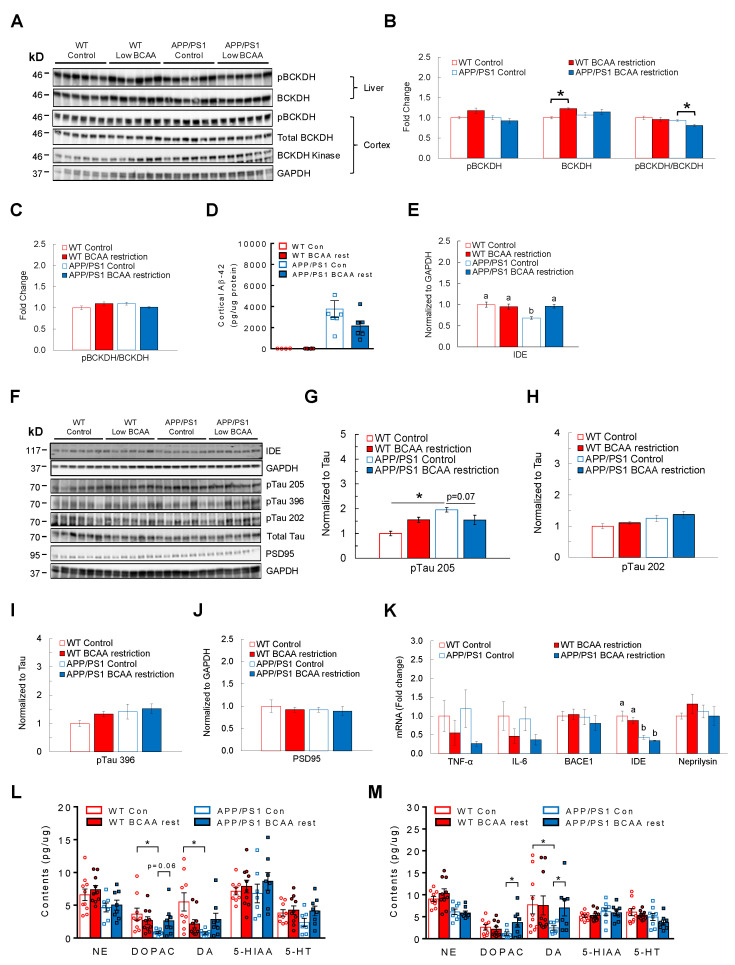
BCAA restriction lowers brain pathology and restores neurotransmitter content in APP/PS1 mice. 11-month-old WT or APP/PS1 mice without cognitive impairment were placed on a control or BCAA-restricted (50%) diet that is iso-caloric and iso-nitrogenous for two months. (**A**) Western blots for proteins involved in BCAA degradation in liver and cortex of the brain. (**B**) pBCKDH and total BCKDH in liver at the end of two months. (**C**) BCKDH inactivity index (pBCKDH/BCKDH) in the cortex. (**D**) Cortical Aβ-42 levels normalized to protein by ELISA. (**E**) Insulin-degrading enzyme (IDE) in the cortex of the brain. (**F**) Western blots for IDE, PSD95, and phosphorylated Tau in the cortex. (**G**) Phosphorylated state of Tau at threonine residue 205, (**H**) Serine residue 202, and (**I**) Serine residue 396. (**J**) Protein expression of PSD95. (**K**) Analysis of genes involved in neuroinflammation and amyloid production and degradation, normalized to B2M. (**L**) Monoamine neurotransmitter concentrations in the hippocampus and (**M**) Cortex. NE—Norepinephrine; DA—Dopamine; DOPAC—Dopamine metabolite; 5-HT—Serotonin; 5-HIAA—Serotonin metabolite. * *p* < 0.05. Groups with different letters are significantly different from each other with *p* < 0.05.

To understand possible mechanisms by which limiting BCAA intake slows down AD development, we focused on examining key markers of AD-related brain pathology and neurotransmitter levels in the hippocampus and cortex as an indicator of neuronal damage and health (Figure 5A). BCAA-restricted APP/PS1 mice had a significantly lower ratio of pBCKDH to BCKDH in liver compared to control diet-fed APP/PS1 mice (main effect of treatment: F(1,20) = 4.38, *p* = 0.049; main effect of genotype: F(1,20) = 8.59, *p* = 0.008; Figure 5B), indicating enhanced hepatic BCAA catabolism that would likely lower circulating BCAAs [42,46]. However, the inactivity index was not different in the cortex across groups (Figure 5C). Next, we assessed amyloid peptide and phosphorylated Tau levels in the cortex as these are considered to be the primary culprits driving AD progression. APP/PS1 mice fed BCAA-restricted diet displayed lower Aβ-42 levels compared to the group on a control diet, although it was not statistically significant (main effect of genotype: F(1,19) = 5.42, *p* = 0.03; Figure 5D). In keeping with this finding, while APP/PS1 controls showed a markedly decreased protein expression of insulin-degrading enzyme (IDE), the enzyme known to break down amyloid peptide, BCAA restriction completely reversed it (main effect of treatment: F(1,20) = 4.89, *p* = 0.039; main effect of genotype: F(1,20) = 8.47, *p* = 0.008; interaction: F(1,20) = 9.87, *p* = 0.005; Figure 5E). Furthermore, limiting BCAA intake tended to reverse the highly phosphorylated state of Tau at Thr205, but not at Ser202 or 396, in APP/PS1 mice compared to those fed a control diet (main effect of genotype: F(1,20) = 14.46, *p* = 0.001; interaction: F(1,20) = 14.72, *p* = 0.001; Figure 5F–I). Contrary to what we expected based on the effects of BCAA supplementation on HT-22 hippocampal neurons, PSD95 protein was not increased in BCAA-restricted APP/PS1 mice compared to control diet-fed counterparts or WTs (Figure 5J). Neuroinflammation induced by microglia is thought to be primarily triggered by soluble Aβ oligomers [66] and play a central role in promoting formation of Tau pathology [67,68,69]. APP/PS1 mice fed BCAA-restricted diet displayed no significant differences in pro-inflammatory markers such as TNF-α and IL-6 and Aβ-degrading enzymes including IDE and neprilysin (Figure 5K). Sufficient neurotransmitter (NT) synthesis or concentration is essential for the overall brain health, and lower monoaminergic neurotransmitters such as norepinephrine (NE), dopamine (DA), and serotonin (5-HT) are observed in AD brains [70,71,72,73]. With more recent studies demonstrating that lower NT levels or degeneration of NT-synthesizing neurons are often observed in the early phase of AD development [72,74,75,76], preventing or reversing the neuronal deterioration is expected to alleviate AD progression and related symptoms. Indeed, control diet-fed APP/PS1 mice showed significantly reduced levels of DA and its metabolite DOPAC, and a non-significant reduction in NE and 5-HT in the hippocampus (DA—main effect of genotype: F(1,30) = 4.50, *p* = 0.042; interaction: F(1,30) = 7.87, *p* = 0.008; NE—main effect of genotype: F(1,31) = 7.67, *p* = 0.009) and cortex (DOPAC—interaction: F(1,30) = 4.32, *p* = 0.046; NE—main effect of genotype: F(1,32) = 23.98, *p* = 0.0001) compared to WTs, but BCAA restriction for two months was able to improve the NT concentrations in both brain regions, most notably DA and DOPAC (Figure 5L,M). Altogether, these data suggest that BCAA restriction effectively lowers amyloid and Tau pathology that is associated with reduced inflammation in the hippocampus of APP/PS1 mice. This is also linked with reinstatement of monoamine neurotransmitters in both cortex and hippocampus, which may in part explain the observed improvement in cognitive function.

### 3.6. BT2, a BCAA-Lowering Compound, Effectively Reduces Aβ-42 and Enhances Cortical and Hippocampal Neurotransmitter Levels in 5xFAD Mice

**Figure 6:**
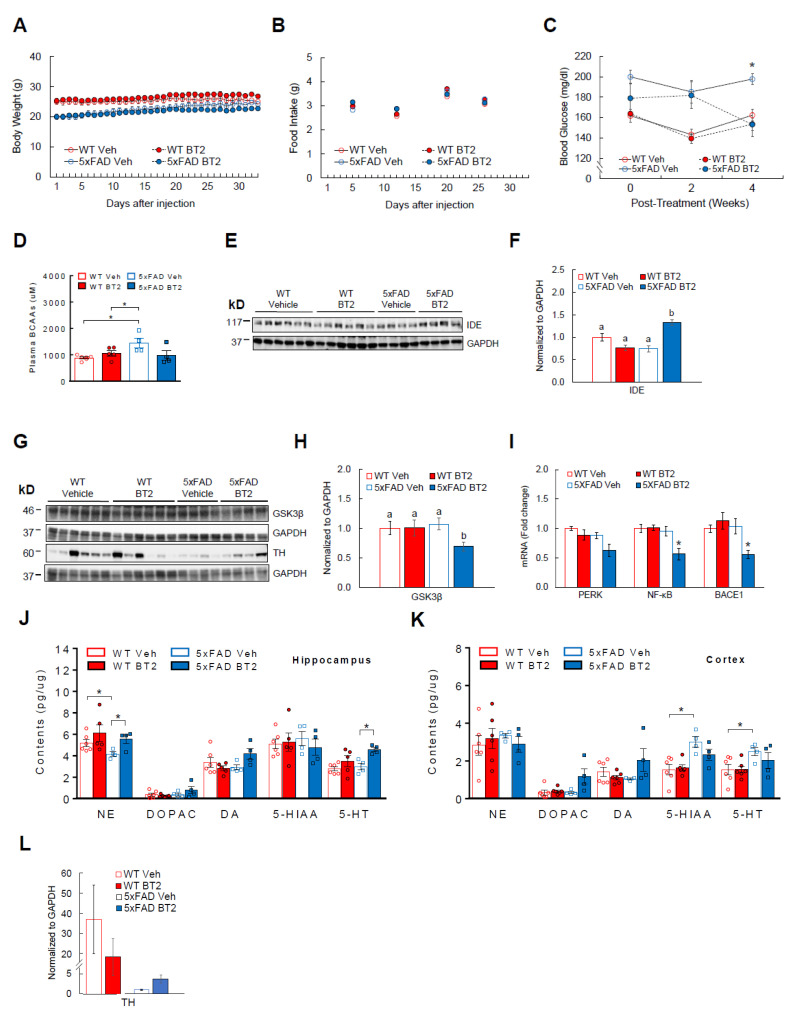
BT2 is effective in alleviating brain pathology and increasing neurotransmitters in 5xFAD mice. WT or 5xFAD mice that are 6–8 weeks old were injected with either vehicle or BT2 (40 mg/kg/day) for one month. Animals were single-housed during the experiment. (**A**) Daily body weight. (**B**) Weekly food intake. (**C**) Blood glucose at baseline and at two and four weeks post-treatment. (**D**) Plasma BCAA levels at baseline and post-treatment. (**E**) Western blots for IDE in the cortex. (**F**) Protein analysis of IDE. (**G**) Western blots for GSK3β in the cortex and tyrosine hydroxylase (TH), the rate-limiting enzyme for catecholamine synthesis, in the hippocampus. (**H**) Protein expression of GSK3β. (**I**) Hippocampal mRNA abundance of markers for ER stress (PERK), inflammation (NF-κB), and amyloid synthesis (BACE1; also known as β-secretase), normalized to B2M. (**J**) Concentration of neurotransmitters (NE, DA, 5-HT) and their metabolites (DOPAC, 5-HIAA) measured in the hippocampus and (**K**) Cortex. (**L**) TH protein expression in the hippocampus. WT vehicle or BT2 group (*n* = 6/group); 5xFAD vehicle or BT2 group (*n* = 4/group). * *p* < 0.05. Groups with different letters are significantly different from each other with *p* < 0.05.

As a complementary strategy to dietary BCAA restriction, we used a pharmacological approach to lower circulating BCAAs and examine the beneficial effects in AD mice. BT2 is an allosteric inhibitor of BCKDH kinase that suppresses BCKDH activity, leading to increased BCAA catabolism and lower plasma BCAA levels [12,15,45,46,47]. For one month, BT2 (40 mg/kg ip) was injected daily to 5xFAD mice, another widely used AD model that overexpresses three APP and two presenilin (PSEN1) mutations [77]. These transgenic mice develop amyloid plaques and neuroinflammation as early as 1.5–2 months of age [77], thus allowing us to investigate the role of BCAAs in early AD-related brain pathology. Body weight (Figure 6A) and food intake (Figure 6B) were not different between WT and 5xFAD mice regardless of treatments. Supporting the data from APP_Swe_ and APP/PS1 mouse experiments, 5xFAD mice showed significantly higher plasma BCAAs compared to WT mice at baseline, but BT2 was able to normalize plasma BCAAs as expected (interaction: F(1,14) = 5.58, *p* = 0.03; Figure 6D). In keeping with the findings in BCAA restriction experiment, cortical BCAA levels were not different between groups (Supplementary Figure S2). 5xFAD mice also had elevated blood glucose compared to WTs before treatment (*p* < 0.05; Figure 6C). However, similar to what was observed in APP/PS1 mice fed BCAA-restriction diet, BT2-treated 5xFAD mice were able to markedly decrease blood glucose compared to vehicle-treated 5xFAD mice (main effect of treatment: F(1,16) = 13.69, *p* = 0.002; main effect of genotype: F(1,16) = 5.95, *p* = 0.027; interaction: F(1,16) = 6.12, *p* = 0.025; Figure 6C). No change was seen within WT groups. Next, we examined AD-related pathology in the cortex. BT2 treatment significantly elevated cortical IDE, an enzyme known to degrade Aβ-42 (70%; main effect of treatment: F(1,16) = 5.25, *p* = 0.035; main effect of genotype: F(1,16) = 4.28, *p* = 0.055; interaction: F(1,16) = 29.33, *p* = 0.0001; Figure 6E,F). In support of this, Tau-phosphorylating and pro-inflammatory enzyme GSK3β was significantly reduced in the cortex after BT2 treatment (Figure 6G,H). In the hippocampus, mRNA of the inflammation mediator (NF-κB) was significantly decreased in BT2-treated 5xFAD mice (main effect of treatment: F(1,15) = 6.46, *p* = 0.022; main effect of genotype: F(1,15) = 10.68, *p* = 0.005; interaction: F(1,15) = 7.23, *p* = 0.016) and the mediator for ER stress (PERK) also showed reduction although not statistically significant (main effect of treatment: F(1,15) = 6.34, *p* = 0.023; main effect of genotype: F(1,15) = 8.86, *p* = 0.019). More interestingly, BACE1 (also known as β-secretase) that is responsible for cleaving APP to promote Aβ-42 production was markedly lowered in these mice (main effect of genotype: F(1,14) = 6.15, *p* = 0.026; interaction: F(1,14) = 7.96, *p* = 0.013; Figure 6I). As in the BCAA dietary restriction experiment, we sought to determine the effects of BT2 on neuronal health by measuring neurotransmitter levels in the hippocampus and cortex. Our data demonstrate that 5xFAD control mice have a significant reduction of NE compared to WT controls in the hippocampus, but NE is fully restored in BT2-treated 5xFAD mice (main effect of treatment: F(1,15) = 5.11, *p* = 0.039; Figure 6J). We also observed a trend of higher DA (40%; *p* = 0.08; interaction: F(1,15) = 5.26, *p* = 0.036) and markedly higher 5-HT (50%; *p* < 0.05; main effect of treatment: F(1,15) = 10.79, *p* = 0.005) after BT2 treatment. Similar increase was shown for DA (180%; interaction: F(1,15) = 4.17, *p* = 0.059) and its metabolite DOPAC (300%; main effect of treatment: F(1,16) = 6.36, *p* = 0.02; main effect of genotype: F(1,16) = 4.48, *p* = 0.05; interaction: F(1,16) = 4.36, *p* = 0.053) in the cortex of BT2-treated 5xFAD mice (Figure 6K), although it was not statistically significant. To confirm greater hippocampal NE levels found in these mice, we assessed protein expression of tyrosine hydroxylase (TH), the rate-limiting enzyme for synthesis of catecholamines including NE and DA. While there was no difference between WT groups, BT2 treatment tended to increase TH by nearly 400% in 5xFAD mice (*p* = 0.07; Figure 6L). Collectively, these results suggest that BT2 recapitulates the pro-neuronal effects of dietary BCAA restriction by lowering AD-related brain pathology and restoring or improving key NT levels in the brain.

## 4. Discussion

Metabolic disorders such as obesity and T2D are associated with a higher incidence of AD [65,78,79,80]. Recent studies suggest that BCAAs are not just associated with obesity and T2D, two strong risk factors for AD, but they also are capable of impairing glycemic control and insulin sensitivity [9,10,11,12,13,14,15]. Moreover, supplementation of BCAAs and/or their metabolites has been shown to induce oxidative stress, apoptosis, neurotransmitter imbalance, and neuronal dysfunctions [16,18,19,21,23]. Given that all these impairments represent key pathological traits shared by AD, this begs the question of whether or not BCAAs may be a contributor to AD pathogenesis. By incorporating experiments with hippocampal HT-22 neurons in vitro, three transgenic AD mouse models in vivo, as well as samples from human AD subjects, we demonstrate that (1) circulating BCAAs and their metabolites are elevated in AD, (2) BCAA supplementation impairs neuronal health and induces AD-related pathology, (3) dietary restriction of BCAAs lowers the brain pathology and alleviates AD progression, and (4) a pharmacological approach to lower BCAAs via BT2 recapitulates the beneficial effects of BCAA restriction.

The role of BCAAs in AD is only recently highlighted due to a growing number of studies linking BCAAs to other AD-related chronic illnesses such as cardiovascular diseases [81,82] and obesity/T2D [4,6,8,52,83]. Studies so far revealed inconsistent findings on the association between circulating BCAAs and AD, with increase [30,35,84], decrease [31,32,37], no change [29,39], or sex-specific [85,86] in AD subjects compared to cognitively healthy controls While the reasons for these conflicting reports are not fully understood, it appears that the findings on BCAAs and AD may vary substantially due to a number of confounding factors such as sex and BMI primarily, but also comorbidities (cardiovascular, endocrine, neurological/psychiatric), medications, ethnicity, severity of AD, and the diagnostic modality of the disease. For instance, in a recent study [40] that included the largest number of participants to date (22,623 Caucasian subjects total from eight different cohorts with incident dementia and AD), Tynkkynen and colleagues demonstrated that lower serum BCAAs are associated with increased risk of dementia and AD. However, as stated by the authors, the association disappeared once the statistical models were adjusted for BMI and cholesterol-lowering medications. With age- and BMI-matched participants without many comorbidities, our metabolomics analysis clearly demonstrates that serum BCAAs and their metabolites are consistently elevated in AD subjects compared to those in healthy individuals, which is in agreement with recent studies [35,84]. While higher circulating BCAA levels are present in people with T2D [4,6,8,52,83], our novel findings revealing even greater serum levels of BCAAs and their metabolites in individuals with AD+T2D compared to those with T2D alone suggest that the relationship between BCAAs and AD extends beyond the effect of diabetes. Considering that obesity or T2D increases the severity of AD progression [65,78,79,80], it is conceivable that having T2D likely amplified BCAA metabolic defect in these individuals during the course of AD development. A controlled longitudinal study that examines circulating BCAAs and their related metabolic signatures in individuals that develop both T2D and AD (in a specific order) would be necessary to test this hypothesis. A robust correlation between a BCAA-derived metabolite and MMSE scores further confirms and strengthens our observations that BCAAs are positively associated with AD and the severity of dementia. Animal models of AD that allow for minimizing many aforementioned confounding variables seem to show a positive relationship between plasma BCAAs and AD. Pan et al. [87] and Ruiz et al. [36] both used transgenic APP/PS1 mouse model and showed higher plasma BCAAs in AD mice compared to age- and weight-matched WTs. The study by Wang and colleagues [84] showed higher plasma isoleucine in 7-month-old 5xFAD mice. In agreement with these findings, in our current study, widely used APP_Swe_ mice (also known as Tg2576) display significantly higher plasma BCAAs compared to WT littermates. Similar to what has been observed in obesity/insulin resistance and T2D [6,12,15,42], these mice show impaired BCAA breakdown in liver that most probably explains a greater BCAA buildup in the circulation. While liver stands as a primary organ that has high capacity to degrade BCAAs [41], other peripheral tissues such as white adipose tissue and muscle may play a role.

Treatment of HT-22 hippocampal neurons with a mixture of BCAAs resulted in a dose-dependent downregulation of genes crucial for healthy neuronal functions including autophagy, mitochondrial biogenesis and fusion, and synapse formation. It is interesting to note that similar impairments are often observed in neurodegenerative diseases such as AD [57,88]. Along with decreased, phosphorylated state of GSK3β (i.e., leading to more active GSK3β) and correspondingly increased pTau, these findings establish a potential causal relationship between BCAAs and AD. Glucose is a versatile nutrient substrate in the brain whose role cannot be fully replaced by any other fuels, thus its metabolic capacity is considered a good indicator of neuronal health that is shown to be impaired in AD [58,59]. A significant reduction of genes involved in the glycolytic pathway in BCAA-treated HT-22 neurons indicates detrimental effects of BCAAs on neuronal glucose metabolism. Given the similar decrease of mRNAs induced by high glucose-induced neurotoxicity, these data further support the concept that BCAAs may substantially impair metabolic/cellular functions and contribute to AD-like neuronal pathology. It is unknown if BCAAs can induce similar AD-like pathological features on neurons in other brain regions affected in AD such as the frontal cortex, hypothalamus, and brainstem [71,72,73,89], or on supporting neural cells including microglia and astrocytes. Glial cells play a major role in keeping micro-environment surveillance and providing essential nutrients and cytoarchitecture support to neurons [90]. They also express BCKDH enzyme and thus are able to catabolize BCAAs [41]. While it is not feasible to examine the effects of BCAAs on glial cells in this experiment since HT-22 cells are an isolated neuronal cell line, it would be both interesting and important to test this in an isolated astrocyte/microglia cell line as well as in a primary mixed neuronal cell culture to understand possible worsening effects of BCAAs on neuronal functions, and whether this is mediated by gliosis and the resultant inflammation and oxidative stress.

BCAAs or amino acids in general cannot be stored in the body for later use as in the case of glucose or lipids, therefore their circulating levels can be only regulated by dietary intake and degradation in tissues [91]. Since we observed impaired hepatic BCAA breakdown and elevated plasma BCAAs in APP_Swe_ mice, we reasoned that changing the other arm of the equation, i.e., dietary intake, is a logical strategy to lower plasma BCAAs in AD. Relevant to this, a growing number of studies suggest that dietary intervention can lower susceptibility to age-related cognitive impairment or AD [92,93,94,95]. In particular, protein restriction has been shown to improve cognitive performance in AD mice [92], although the role of BCAAs in this limited protein intake is unknown. A recent study by Tournissac and others [63] demonstrated that BCAA manipulation is effective in improving memory and lowering pTau deposits in 3xTg-AD mice on a high-fat (HF) diet. The interpretation of the results, however, is unclear because of the lack of age-matched, healthy WT control mice along with the use of diets with different amounts of nitrogen levels. In the current study, we fed animals either a purified control diet or a customized control diet that is restricted of all three BCAAs while being iso-caloric and iso-nitrogenous. We considered this to be an important control variable since changes in the caloric content or the amount of nitrogen source would have a substantial impact on energy balance, protein or nucleic acid synthesis, and enzymatic activity for metabolism, among other physiological adaptations. While maintaining negligible changes in the amount of most amino acids, we made sure that amino acids that compete with BCAAs (tyrosine, tryptophan, and phenylalanine) are not altered between the diets. Here, we were able to demonstrate that BCAA-restricted diet for just two months substantially ameliorated AD-related brain pathology and cognitive decline in APP/PS1 mice that is independent of changes in body weight or food intake. This was associated with reduced blood glucose and inflammatory mediators in the hippocampus. Impaired glucose homeostasis or insulin resistance, as commonly observed in T2D patients, often precedes AD-related brain pathology and/or cognitive deficits [96,97,98,99,100,101,102]. Given that this pre-diabetic impairment in systemic glucose control and insulin sensitivity can potentially contribute to the pathogenesis of AD, our results suggest that a simple dietary intervention of limiting BCAA intake is effective in not only reducing amyloid and Tau burden in the brain, but also in correcting metabolic dysregulation. Whether or not the improved glycemic control following BCAA restriction in APP/PS1 mice is in part responsible for reduced brain pathology and delayed memory loss needs to be explored in the future.

Both BCAA restriction and BT2 treatment significantly improved monoamine neurotransmitter levels in the hippocampus and cortex of APP/PS1 and 5xFAD mice, respectively, hence demonstrating profound pro-neuronal effects of BCAA reduction. Neurotransmitter concentration is determined by both synthesis and degradation. Both treatments most likely did not affect the synthesis since we failed to find any differences in both protein expression of tyrosine hydroxylase (rate-limiting enzyme for catecholamines), dopamine-beta hydroxylase (enzyme responsible for NE synthesis), or tryptophan hydroxylase (rate-limiting enzyme for 5-HT synthesis) in the cortex and mRNA expression of these enzymes in the brainstem (not shown). It is possible that the enhanced cortical and hippocampal neurotransmitter levels in these AD mice are in part attributed to decreased neurotransmitter breakdown by monoamine oxidase enzymes present around the synapse. This warrants further investigation. We also acknowledge that measuring monoamine concentrations at any one timepoint provides only a static measurement of neurotransmitter activity. A more involved method such as microdialysis or push-pull perfusion might provide a more dynamic measure of how neurotransmitters are released in these animals. Notably, 5xFAD transgenic mice are phenotypically different from APP/PS1 mice [103,104] in that without forming Tau aggregates, they develop amyloid pathology early at six weeks of age and spatial memory impairment when they are about four months old [77,105], thus reflecting relatively expedited progression of AD. Our results demonstrating a clear alleviation of brain pathology and restored neurotransmitter levels in these two popular, but distinct AD mouse models further strengthen our hypothesis that either dietary or pharmacological strategy to lower circulating BCAAs is effective in treating and/or delaying AD progression. It would be important in the future to test different doses and administration routes to refine the efficacy of BT2 in treating AD. Unexpectedly, unlike 5xFAD mice, WT mice did not respond to BT2 by lowering plasma BCAAs. Although the reasons are unclear, the lack of decrease in BCAAs was also observed in a previous study [45]. We speculate that this may be partly due to the specific strain of WT mice with C57Bl/6 × SJL background since we have observed lowered plasma BCAAs following BT2 injection in regular C57Bl/6J mice.

While plasma BCAAs at the end of BCAA restriction experiment could not be measured due to heavy hemolysis of trunk blood, we believe that they are most probably lowered in BCAA-restricted APP/PS1 mice as expected, in parallel to the reduction observed in BT2-treated 5xFAD mice (Figure 6D), for the following reasons. First, both BCAA restriction and BT2 with the same 50% restriction and the dose, respectively, have been shown by others to significantly lower plasma BCAAs and this led to enhanced glucose homeostasis and insulin sensitivity in either lean, diet-induced obese, or genetically diabetic (db/db) rats and mice [6,9,10,12,15]. Similarly, both mouse models in our study (APP/PS1, 5xFAD) displayed a marked improvement in glycemic control. Second, Tournissac and others [63] have recently demonstrated a substantial reduction of plasma BCAAs in 3xTg AD mice treated with BCAA restriction diet (same 50% restriction). Third, BCAA-restricted APP/PS1 mice displayed significantly higher BCAA breakdown in liver as evidenced by lower inactivity index (pBCKDH/BCKDH), pointing to lower circulating BCAAs. Fourth, since BCAAs compete with neurotransmitter precursors (i.e., aromatic amino acids such as tyrosine, phenylalanine, tryptophan) to enter the brain via the same large amino acid transporter (LAT) [106], it is hypothesized that by gaining access to the brain, high plasma BCAAs interfere with the entry of precursors that would dramatically reduce neurotransmitter synthesis as observed in AD. Our data showing restored monoamine neurotransmitters after BCAA restriction or BT2 treatment suggest that this is most likely mediated by lowering of plasma BCAAs that would allow more entry of precursors for neurotransmitter synthesis into the brain.

Our study has several limitations. Seven AD patients and five patients suffering from AD and T2D were on donepezil or memantine (medications for AD), whereas three age-matched, health controls have taken meloxicam (medication for arthritis). Like other studies described above, it is possible that even this small number of prescribed medications and the pharmacological nature of them may have influenced the outcomes we observed. Also, our results cannot be applied to women with AD since we did not include females in our study. Because on average women are more affected by AD than men, we believe it is critical to investigate the role of BCAAs in women with AD and determine if there are any sex differences in a more controlled setting that minimizes aforementioned confounding variables, as well as to test the efficacy of BT2 in female AD subjects.

In conclusion, our findings shed light on the potential role of BCAAs in AD pathophysiology by demonstrating not only a consistent positive association in both humans and three different rodent AD models, but also a potential causal effect of BCAAs on AD-related neuropathology. Both dietary and pharmacological approaches that lower circulating BCAAs reveal similar and promising results in alleviating AD-related pathology and cognitive decline. Further studies are needed to understand when the disease-modifying effects of BCAA reduction is most optimal during AD development, and if the effects are in large peripherally or centrally mediated.

## Figures and Tables

**Table 1:** Detailed nutrient composition in Control diet and customized, 50% BCAA-restricted diet.

	Control Diet Research Diets A11072001		(50%) BCAA-Restricted Diet Research Diets A20112501	
Macronutrients	gm	kcal	gm	kcal
Protein	34	33	34	33
Carbohydrate	49	49	49	49
Fat	8	18	8	18
Fiber	4	0	4	0
Total	91	100	91	100
kcal/gm	4		4	
**Amino Acids**				
Cystine	5.5	22	6.29	25
Isoleucine	17.88	72	8.94	36
Leucine	31.63	127	15.81	63
Lysine	24.75	99	29.28	117
Methionine	6.88	28	7.86	31
Phenylalanine	17.88	72	17.88	72
Threonine	15.13	61	17.29	69
Tryptophan	5.5	22	5.50	22
Valine	23.38	94	11.69	47
Histidine	8.25	33	9.43	38
Alanine	12.38	50	14.15	57
Arginine	27.5	110	32.08	128
Aspartic Acid	20.63	83	23.58	94
Glutamic Acid	37.13	149	43.43	174
Glycine	38.5	154	45.00	180
Proline	17.88	72	20.43	82
Serine	15.13	61	17.29	69
Tyrosine	12.38	50	12.38	50
**Other Ingredients**				
Corn Starch	386	1544	386	1544
Maltodextrin 10	100	400	100	400
Cellulose, BW200	40	0	40	0
Soybean Oil	25	225	25	225
Lard	55	495	55	495
Mineral Mix S10026	10	0	10	0
Dicalcium Phosphate	13	0	13	0
Calcium Carbonate	5.5	0	6	0
Potassium Citrate, 1 H_2_O	16.5	0	17	0
Sodium Bicarbonate	3.5	0	3.5	0
Vitamin Mix V10001	10	40	10	40
Choline Bitartrate	2	0	2	0
Cholesterol	0.782	0	0.782	0
**Total**	**1005.64**	**4057**	**1005.592**	**4057**

**Table 2:** Primer sequences for genes measured by RT-qPCR.

Gene Name		Primer Sequence (5′–3′)
BCKDH	Forward	GGATGAGGAACAGGAGAAGG
	Reverse	GGAGAAGAGGAGGCTTGG
BCKDH Kinase	Forward	GACAGGTGGACTTAGATGGA
	Reverse	CAAGAATGAGCAGAGCAGAG
BCKDH Phosphatase	Forward	CCTGCTACTTCTCCACTTCA
	Reverse	GCTCATCAATGCGGTTATCC
LC3A	Forward	CCCATCGCTGACATCTATGAAC
	Reverse	AAGGTTTCTTGGGAGGCGTA
Beclin1	Forward	ACCAGCGGGAGTATAGTGAGT
	Reverse	CAGCTGGATCTGGGCGTAG
Nrf1	Forward	AGAAACGGAAACGGCCTCAT
	Reverse	CATCCAACGTGGCTCTGAGT
Nrf2	Forward	ATGGAGCAAGTTTGGCAGGA
	Reverse	GCTGGGAACAGCGGTAGTAT
PSD95	Forward	CTTCATCCTTGCTGGGGGTC
	Reverse	TTGCGGAGGTCAACACCATT
Opa1	Forward	ACCTTGCCAGTTTAGCTCCC
	Reverse	TTGGGACCTGCAGTGAAGAA
Mfn1	Forward	GCAGACAGCACATGGAGAGA
	Reverse	GATCCGATTCCGAGCTTCCG
Mfn2	Forward	TGCACCGCCATATAGAGGAAG
	Reverse	TCTGCAGTGAACTGGCAATG
VPS26	Forward	CCAGCCGAAGTGTCCATA
	Reverse	CCATACGCCTCAGTTGTG
VPS36	Forward	ACCTCCAGACACCTTCAG
	Reverse	CTCCATTAGTAGCCAGAATAAGTG
Hexokinase	Forward	TGCCACTGAGTTGTCTGT
	Reverse	CTACCACCACCACCATCA
TPI	Forward	CAGCAGGCACAGGAAGTA
	Reverse	CCAGTCACAGAACCTCCATAA
Enolase	Forward	CACAGTTGCCACCATCTC
	Reverse	TTCTCTTCGTCCTCTCACAT
Aconitase	Forward	AGATACGGACGCTTACCATT
	Reverse	CGGCACTTCTATGTTCTTATGTT
Pyruvate Kinase	Forward	CGCAACACTGGCATCATT
	Reverse	TGGCTTCACGGACATTCT
B2M	Forward	GAAGCCGAACATACTGAACTG
	Reverse	CTGAAGGACATATCTGACATCTCT
GAPDH	Forward	GGTGAAGGTCGGTGTGAAC
	Reverse	TGAGTGGAGTCATACTGGAACA
BACE1	Forward	CCTATGCGATGCGAATGTT
	Reverse	TCTCCTTCCTGTCTCTATCCT
PSEN1	Forward	CACCGTTGTCCTACTTCCA
	Reverse	CTCCTCATCTTCTTCCTCATCTT
PERK	Forward	ACGGTTACTATCTGCCATACTAC
	Reverse	CCTTCTTGCGGATGTTCTTG
TNF-α	Forward	ACCACCATCAAGGACTCAA
	Reverse	AAGGTCTGAAGGTAGGAAGG
IL-6	Forward	ACAGAAGGAGTGGCTAAG
	Reverse	AGAGAACAACATAAGTCAGATAC

## Data Availability

The data that support the findings of this study are not publicly available due to propriety reasons, but are available from the corresponding author upon reasonable request.

## Supplementary Materials

The following supporting information can be downloaded at: https://www.mdpi.com/article/10.3390/cells11213523/s1. Table S1: Patient information and exclusion characteristics. Table S2: Fold changes of serum metabolites between AD and age-matched healthy controls. Figure S1: BCAA levels measured in the neocortex of WT and APP/PS1 mice with control diet or BCAA-restricted diet at the end of two months. Figure S2: BCAA levels measured in the neocortex of WT and 5xFAD mice with 30-day treatment of either vehicle or BT2.

## References

[1] Alzheimer’s Association Alzheimer’s Disease Facts and Figures. https://www.alz.org/alzheimers-dementia/facts-figures.

[2] Salas I.H., De Strooper B. (2019). Diabetes and Alzheimer’s Disease: A Link not as Simple as it Seems. Neurochem. Res..

[3] Umegaki H. (2014). Type 2 diabetes as a risk factor for cognitive impairment: Current insights. Clin. Interv. Aging.

[4] Adams S.H., Hoppel C.L., Lok K.H., Zhao L., Wong S.W., Minkler P.E., Hwang D.H., Newman J.W., Garvey W.T. (2009). Plasma acylcarnitine profiles suggest incomplete long-chain fatty acid beta-oxidation and altered tricarboxylic acid cycle activity in type 2 diabetic African-American women. J. Nutr..

[5] Mihalik S.J., Goodpaster B.H., Kelley D.E., Chace D.H., Vockley J., Toledo F.G., DeLany J.P. (2010). Increased levels of plasma acylcarnitines in obesity and type 2 diabetes and identification of a marker of glucolipotoxicity. Obesity.

[6] Newgard C.B., An J., Bain J.R., Muehlbauer M.J., Stevens R.D., Lien L.F., Haqq A.M., Shah S.H., Arlotto M., Slentz C.A. (2009). A branched-chain amino acid-related metabolic signature that differentiates obese and lean humans and contributes to insulin resistance. Cell Metab..

[7] She P., Van Horn C., Reid T., Hutson S.M., Cooney R.N., Lynch C.J. (2007). Obesity-related elevations in plasma leucine are associated with alterations in enzymes involved in branched-chain amino acid metabolism. Am. J. Physiol. Endocrinol. Metab..

[8] Wang T.J., Larson M.G., Vasan R.S., Cheng S., Rhee E.P., McCabe E., Lewis G.D., Fox C.S., Jacques P.F., Fernandez C. (2011). Metabolite profiles and the risk of developing diabetes. Nat. Med..

[9] Cummings N.E., Williams E.M., Kasza I., Konon E.N., Schaid M.D., Schmidt B.A., Poudel C., Sherman D.S., Yu D., Arriola Apelo S.I. (2018). Restoration of metabolic health by decreased consumption of branched-chain amino acids. J. Physiol..

[10] Fontana L., Cummings N.E., Arriola Apelo S.I., Neuman J.C., Kasza I., Schmidt B.A., Cava E., Spelta F., Tosti V., Syed F.A. (2016). Decreased Consumption of Branched-Chain Amino Acids Improves Metabolic Health. Cell Rep..

[11] Moghei M., Tavajohi-Fini P., Beatty B., Adegoke O.A. (2016). Ketoisocaproic acid, a metabolite of leucine, suppresses insulin-stimulated glucose transport in skeletal muscle cells in a BCAT2-dependent manner. Am. J. Physiol. Cell Physiol..

[12] White P.J., Lapworth A.L., An J., Wang L., McGarrah R.W., Stevens R.D., Ilkayeva O., George T., Muehlbauer M.J., Bain J.R. (2016). Branched-chain amino acid restriction in Zucker-fatty rats improves muscle insulin sensitivity by enhancing efficiency of fatty acid oxidation and acyl-glycine export. Mol. Metab..

[13] Xiao F., Yu J., Guo Y., Deng J., Li K., Du Y., Chen S., Zhu J., Sheng H., Guo F. (2014). Effects of individual branched-chain amino acids deprivation on insulin sensitivity and glucose metabolism in mice. Metabolism.

[14] Jang C., Oh S.F., Wada S., Rowe G.C., Liu L., Chan M.C., Rhee J., Hoshino A., Kim B., Ibrahim A. (2016). A branched-chain amino acid metabolite drives vascular fatty acid transport and causes insulin resistance. Nat. Med..

[15] Zhou M., Shao J., Wu C.Y., Shu L., Dong W., Liu Y., Chen M., Wynn R.M., Wang J., Gui W.J. (2019). Targeting BCAA Catabolism to Treat Obesity-Associated Insulin Resistance. Diabetes.

[16] Bridi R., Braun C.A., Zorzi G.K., Wannmacher C.M., Wajner M., Lissi E.G., Dutra-Filho C.S. (2005). alpha-keto acids accumulating in maple syrup urine disease stimulate lipid peroxidation and reduce antioxidant defences in cerebral cortex from young rats. Metab. Brain Dis..

[17] Lu G., Sun H., She P., Youn J.Y., Warburton S., Ping P., Vondriska T.M., Cai H., Lynch C.J., Wang Y. (2009). Protein phosphatase 2Cm is a critical regulator of branched-chain amino acid catabolism in mice and cultured cells. J. Clin. Investig..

[18] Amaral A.U., Leipnitz G., Fernandes C.G., Seminotti B., Schuck P.F., Wajner M. (2010). Alpha-ketoisocaproic acid and leucine provoke mitochondrial bioenergetic dysfunction in rat brain. Brain Res..

[19] Lu G., Ren S., Korge P., Choi J., Dong Y., Weiss J., Koehler C., Chen J.N., Wang Y. (2007). A novel mitochondrial matrix serine/threonine protein phosphatase regulates the mitochondria permeability transition pore and is essential for cellular survival and development. Genes Dev..

[20] Haymond M.W., Ben-Galim E., Strobel K.E. (1978). Glucose and alanine metabolism in children with maple syrup urine disease. J. Clin. Investig..

[21] Snyderman S.E., Goldstein F., Sansaricq C., Norton P.M. (1984). The relationship between the branched chain amino acids and their alpha-ketoacids in maple syrup urine disease. Pediatr. Res..

[22] Brosnan J.T., Brosnan M.E. (2006). Branched-chain amino acids: Enzyme and substrate regulation. J. Nutr..

[23] Fernstrom J.D. (2005). Branched-chain amino acids and brain function. J. Nutr..

[24] Jeganathan S., Abdullahi A., Zargar S., Maeda N., Riddell M.C., Adegoke O.A. (2014). Amino acid-induced impairment of insulin sensitivity in healthy and obese rats is reversible. Physiol. Rep..

[25] Ono H., Pocai A., Wang Y., Sakoda H., Asano T., Backer J.M., Schwartz G.J., Rossetti L. (2008). Activation of hypothalamic S6 kinase mediates diet-induced hepatic insulin resistance in rats. J. Clin. Investig..

[26] Lee H.K., Kwon B., Lemere C.A., de la Monte S., Itamura K., Ha A.Y., Querfurth H.W. (2017). mTORC2 (Rictor) in Alzheimer’s Disease and Reversal of Amyloid-beta Expression-Induced Insulin Resistance and Toxicity in Rat Primary Cortical Neurons. J. Alzheimers Dis..

[27] Caccamo A., Belfiore R., Oddo S. (2018). Genetically reducing mTOR signaling rescues central insulin dysregulation in a mouse model of Alzheimer’s disease. Neurobiol. Aging.

[28] Li F., Yin Y., Tan B., Kong X., Wu G. (2011). Leucine nutrition in animals and humans: mTOR signaling and beyond. Amino Acids.

[29] Basun H., Forssell L.G., Almkvist O., Cowburn R.F., Eklof R., Winblad B., Wetterberg L. (1990). Amino acid concentrations in cerebrospinal fluid and plasma in Alzheimer’s disease and healthy control subjects. J. Neural. Transm. Park. Dis Dement. Sect..

[30] Fonteh A.N., Harrington R.J., Tsai A., Liao P., Harrington M.G. (2007). Free amino acid and dipeptide changes in the body fluids from Alzheimer’s disease subjects. Amino Acids.

[31] Gonzalez-Dominguez R., Garcia-Barrera T., Gomez-Ariza J.L. (2015). Metabolite profiling for the identification of altered metabolic pathways in Alzheimer’s disease. J. Pharm. Biomed. Anal..

[32] Horgusluoglu E., Neff R., Song W.M., Wang M., Wang Q., Arnold M., Krumsiek J., Galindo-Prieto B., Ming C., Nho K. (2022). Integrative metabolomics-genomics approach reveals key metabolic pathways and regulators of Alzheimer’s disease. Alzheimers Dement..

[33] Ibanez C., Simo C., Martin-Alvarez P.J., Kivipelto M., Winblad B., Cedazo-Minguez A., Cifuentes A. (2012). Toward a predictive model of Alzheimer’s disease progression using capillary electrophoresis-mass spectrometry metabolomics. Anal. Chem..

[34] Larsson S.C., Markus H.S. (2017). Branched-chain amino acids and Alzheimer’s disease: A Mendelian randomization analysis. Sci. Rep..

[35] Li H., Ye D., Xie W., Hua F., Yang Y., Wu J., Gu A., Ren Y., Mao K. (2018). Defect of branched-chain amino acid metabolism promotes the development of Alzheimer’s disease by targeting the mTOR signaling. Biosci. Rep..

[36] Ruiz H.H., Chi T., Shin A.C., Lindtner C., Hsieh W., Ehrlich M., Gandy S., Buettner C. (2016). Increased susceptibility to metabolic dysregulation in a mouse model of Alzheimer’s disease is associated with impaired hypothalamic insulin signaling and elevated BCAA levels. Alzheimers Dement..

[37] Toledo J.B., Arnold M., Kastenmuller G., Chang R., Baillie R.A., Han X., Thambisetty M., Tenenbaum J.D., Suhre K., Thompson J.W. (2017). Metabolic network failures in Alzheimer’s disease: A biochemical road map. Alzheimers Dement..

[38] Tondo M., Wasek B., Escola-Gil J.C., de Gonzalo-Calvo D., Harmon C., Arning E., Bottiglieri T. (2020). Altered Brain Metabolome Is Associated with Memory Impairment in the rTg4510 Mouse Model of Tauopathy. Metabolites.

[39] Trushina E., Dutta T., Persson X.M., Mielke M.M., Petersen R.C. (2013). Identification of altered metabolic pathways in plasma and CSF in mild cognitive impairment and Alzheimer’s disease using metabolomics. PLoS ONE.

[40] Tynkkynen J., Chouraki V., van der Lee S.J., Hernesniemi J., Yang Q., Li S., Beiser A., Larson M.G., Saaksjarvi K., Shipley M.J. (2018). Association of branched-chain amino acids and other circulating metabolites with risk of incident dementia and Alzheimer’s disease: A prospective study in eight cohorts. Alzheimers Dement..

[41] Hutson S.M., Sweatt A.J., Lanoue K.F. (2005). Branched-chain amino acid metabolism: Implications for establishing safe intakes. J. Nutr..

[42] Shin A.C., Fasshauer M., Filatova N., Grundell L.A., Zielinski E., Zhou J.Y., Scherer T., Lindtner C., White P.J., Lapworth A.L. (2014). Brain insulin lowers circulating BCAA levels by inducing hepatic BCAA catabolism. Cell Metab..

[43] Waring S., O’Bryant S.E., Reisch J.S., Diaz-Arrastia R., Knebel J., Doody R. (2008). The Texas Alzheimer’s Research Consortium longitudinal research cohort: Study design and baseline characteristics. Tex. Public Health J..

[44] McKhann G., Drachman D., Folstein M., Katzman R., Price D., Stadlan E.M. (1984). Clinical diagnosis of Alzheimer’s disease: Report of the NINCDS-ADRDA Work Group under the auspices of Department of Health and Human Services Task Force on Alzheimer’s Disease. Neurology.

[45] Sun H., Olson K.C., Gao C., Prosdocimo D.A., Zhou M., Wang Z., Jeyaraj D., Youn J.Y., Ren S., Liu Y. (2016). Catabolic Defect of Branched-Chain Amino Acids Promotes Heart Failure. Circulation.

[46] Tso S.C., Gui W.J., Wu C.Y., Chuang J.L., Qi X., Skvora K.J., Dork K., Wallace A.L., Morlock L.K., Lee B.H. (2014). Benzothiophene carboxylate derivatives as novel allosteric inhibitors of branched-chain alpha-ketoacid dehydrogenase kinase. J. Biol. Chem..

[47] Uddin G.M., Zhang L., Shah S., Fukushima A., Wagg C.S., Gopal K., Al Batran R., Pherwani S., Ho K.L., Boisvenue J. (2019). Impaired branched chain amino acid oxidation contributes to cardiac insulin resistance in heart failure. Cardiovasc. Diabetol..

[48] Wessels A.G., Kluge H., Hirche F., Kiowski A., Schutkowski A., Corrent E., Bartelt J., Konig B., Stangl G.I. (2016). High Leucine Diets Stimulate Cerebral Branched-Chain Amino Acid Degradation and Modify Serotonin and Ketone Body Concentrations in a Pig Model. PLoS ONE.

[49] Purpera M.N., Shen L., Taghavi M., Munzberg H., Martin R.J., Hutson S.M., Morrison C.D. (2012). Impaired branched chain amino acid metabolism alters feeding behavior and increases orexigenic neuropeptide expression in the hypothalamus. J. Endocrinol..

[50] Laeger T., Reed S.D., Henagan T.M., Fernandez D.H., Taghavi M., Addington A., Munzberg H., Martin R.J., Hutson S.M., Morrison C.D. (2014). Leucine acts in the brain to suppress food intake but does not function as a physiological signal of low dietary protein. Am. J. Physiol. Regul. Integr. Comp. Physiol..

[51] Beckett P.R. (2000). Spectrophotometric assay for measuring branched-chain amino acids. Methods Enzymol..

[52] Wurtz P., Soininen P., Kangas A.J., Ronnemaa T., Lehtimaki T., Kahonen M., Viikari J.S., Raitakari O.T., Ala-Korpela M. (2013). Branched-chain and aromatic amino acids are predictors of insulin resistance in young adults. Diabetes Care.

[53] Jankowsky J.L., Zheng H. (2017). Practical considerations for choosing a mouse model of Alzheimer’s disease. Mol. Neurodegener..

[54] Hsiao K., Chapman P., Nilsen S., Eckman C., Harigaya Y., Younkin S., Yang F., Cole G. (1996). Correlative memory deficits, Abeta elevation, and amyloid plaques in transgenic mice. Science.

[55] Lin M.T., Beal M.F. (2006). Mitochondrial dysfunction and oxidative stress in neurodegenerative diseases. Nature.

[56] Mao P., Reddy P.H. (2011). Aging and amyloid beta-induced oxidative DNA damage and mitochondrial dysfunction in Alzheimer’s disease: Implications for early intervention and therapeutics. Biochim. Biophys. Acta.

[57] Yin F., Sancheti H., Patil I., Cadenas E. (2016). Energy metabolism and inflammation in brain aging and Alzheimer’s disease. Free Radic. Biol. Med..

[58] Theurey P., Connolly N.M.C., Fortunati I., Basso E., Lauwen S., Ferrante C., Moreira Pinho C., Joselin A., Gioran A., Bano D. (2019). Systems biology identifies preserved integrity but impaired metabolism of mitochondria due to a glycolytic defect in Alzheimer’s disease neurons. Aging Cell.

[59] Iwangoff P., Armbruster R., Enz A., Meier-Ruge W. (1980). Glycolytic enzymes from human autoptic brain cortex: Normal aged and demented cases. Mech. Ageing Dev..

[60] Tomlinson D.R., Gardiner N.J. (2008). Glucose neurotoxicity. Nat. Rev. Neurosci..

[61] Liu D., Zhang H., Gu W., Liu Y., Zhang M. (2013). Neuroprotective effects of ginsenoside Rb1 on high glucose-induced neurotoxicity in primary cultured rat hippocampal neurons. PLoS ONE.

[62] Russell J.W., Golovoy D., Vincent A.M., Mahendru P., Olzmann J.A., Mentzer A., Feldman E.L. (2002). High glucose-induced oxidative stress and mitochondrial dysfunction in neurons. FASEB J..

[63] Tournissac M., Vandal M., Tremblay C., Bourassa P., Vancassel S., Emond V., Gangloff A., Calon F. (2018). Dietary intake of branched-chain amino acids in a mouse model of Alzheimer’s disease: Effects on survival, behavior, and neuropathology. Alzheimers Dement..

[64] Leibson C.L., Rocca W.A., Hanson V.A., Cha R., Kokmen E., O’Brien P.C., Palumbo P.J. (1997). Risk of dementia among persons with diabetes mellitus: A population-based cohort study. Am. J. Epidemiol..

[65] Ott A., Stolk R.P., Hofman A., van Harskamp F., Grobbee D.E., Breteler M.M. (1996). Association of diabetes mellitus and dementia: The Rotterdam Study. Diabetologia.

[66] Heneka M.T., Carson M.J., El Khoury J., Landreth G.E., Brosseron F., Feinstein D.L., Jacobs A.H., Wyss-Coray T., Vitorica J., Ransohoff R.M. (2015). Neuroinflammation in Alzheimer’s disease. Lancet Neurol..

[67] Maphis N., Xu G., Kokiko-Cochran O.N., Jiang S., Cardona A., Ransohoff R.M., Lamb B.T., Bhaskar K. (2015). Reactive microglia drive tau pathology and contribute to the spreading of pathological tau in the brain. Brain.

[68] Yoshiyama Y., Higuchi M., Zhang B., Huang S.M., Iwata N., Saido T.C., Maeda J., Suhara T., Trojanowski J.Q., Lee V.M. (2007). Synapse loss and microglial activation precede tangles in a P301S tauopathy mouse model. Neuron.

[69] Ising C., Venegas C., Zhang S., Scheiblich H., Schmidt S.V., Vieira-Saecker A., Schwartz S., Albasset S., McManus R.M., Tejera D. (2019). NLRP3 inflammasome activation drives tau pathology. Nature.

[70] Gannon M., Che P., Chen Y., Jiao K., Roberson E.D., Wang Q. (2015). Noradrenergic dysfunction in Alzheimer’s disease. Front. Neurosci..

[71] Liu Y., Yoo M.J., Savonenko A., Stirling W., Price D.L., Borchelt D.R., Mamounas L., Lyons W.E., Blue M.E., Lee M.K. (2008). Amyloid pathology is associated with progressive monoaminergic neurodegeneration in a transgenic mouse model of Alzheimer’s disease. J. Neurosci..

[72] Nobili A., Latagliata E.C., Viscomi M.T., Cavallucci V., Cutuli D., Giacovazzo G., Krashia P., Rizzo F.R., Marino R., Federici M. (2017). Dopamine neuronal loss contributes to memory and reward dysfunction in a model of Alzheimer’s disease. Nat. Commun..

[73] Rorabaugh J.M., Chalermpalanupap T., Botz-Zapp C.A., Fu V.M., Lembeck N.A., Cohen R.M., Weinshenker D. (2017). Chemogenetic locus coeruleus activation restores reversal learning in a rat model of Alzheimer’s disease. Brain.

[74] Braak H., Del Tredici K. (2012). Where, when, and in what form does sporadic Alzheimer’s disease begin?. Curr. Opin Neurol..

[75] Mather M., Harley C.W. (2016). The Locus Coeruleus: Essential for Maintaining Cognitive Function and the Aging Brain. Trends Cogn. Sci..

[76] Smith G.S., Barrett F.S., Joo J.H., Nassery N., Savonenko A., Sodums D.J., Marano C.M., Munro C.A., Brandt J., Kraut M.A. (2017). Molecular imaging of serotonin degeneration in mild cognitive impairment. Neurobiol. Dis..

[77] Oakley H., Cole S.L., Logan S., Maus E., Shao P., Craft J., Guillozet-Bongaarts A., Ohno M., Disterhoft J., Van Eldik L. (2006). Intraneuronal beta-amyloid aggregates, neurodegeneration, and neuron loss in transgenic mice with five familial Alzheimer’s disease mutations: Potential factors in amyloid plaque formation. J. Neurosci..

[78] Bedse G., Di Domenico F., Serviddio G., Cassano T. (2015). Aberrant insulin signaling in Alzheimer’s disease: Current knowledge. Front. Neurosci..

[79] Steen E., Terry B.M., Rivera E.J., Cannon J.L., Neely T.R., Tavares R., Xu X.J., Wands J.R., de la Monte S.M. (2005). Impaired insulin and insulin-like growth factor expression and signaling mechanisms in Alzheimer’s disease--is this type 3 diabetes?. J. Alzheimers Dis..

[80] Yannakoulia M., Kontogianni M., Scarmeas N. (2015). Cognitive health and Mediterranean diet: Just diet or lifestyle pattern?. Ageing Res. Rev..

[81] De Toledo Ferraz Alves T.C., Ferreira L.K., Wajngarten M., Busatto G.F. (2010). Cardiac disorders as risk factors for Alzheimer’s disease. J. Alzheimers Dis.

[82] Santos C.Y., Snyder P.J., Wu W.C., Zhang M., Echeverria A., Alber J. (2017). Pathophysiologic relationship between Alzheimer’s disease, cerebrovascular disease, and cardiovascular risk: A review and synthesis. Alzheimers Dement..

[83] Kim J.Y., Park J.Y., Kim O.Y., Ham B.M., Kim H.J., Kwon D.Y., Jang Y., Lee J.H. (2010). Metabolic profiling of plasma in overweight/obese and lean men using ultra performance liquid chromatography and Q-TOF mass spectrometry (UPLC-Q-TOF MS). J. Proteome Res..

[84] Wang X., Sun G., Feng T., Zhang J., Huang X., Wang T., Xie Z., Chu X., Yang J., Wang H. (2019). Sodium oligomannate therapeutically remodels gut microbiota and suppresses gut bacterial amino acids-shaped neuroinflammation to inhibit Alzheimer’s disease progression. Cell Res..

[85] Zarzar T.G., Lee B., Coughlin R., Kim D., Shen L., Hall M.A. (2022). Sex Differences in the Metabolome of Alzheimer’s Disease Progression. Front. Radiol..

[86] Arnold M., Nho K., Kueider-Paisley A., Massaro T., Huynh K., Brauner B., MahmoudianDehkordi S., Louie G., Moseley M.A., Thompson J.W. (2020). Sex and APOE epsilon4 genotype modify the Alzheimer’s disease serum metabolome. Nat. Commun..

[87] Pan X., Nasaruddin M.B., Elliott C.T., McGuinness B., Passmore A.P., Kehoe P.G., Holscher C., McClean P.L., Graham S.F., Green B.D. (2016). Alzheimer’s disease-like pathology has transient effects on the brain and blood metabolome. Neurobiol. Aging.

[88] Kumar K., Kumar A., Keegan R.M., Deshmukh R. (2018). Recent advances in the neurobiology and neuropharmacology of Alzheimer’s disease. Biomed. Pharmacother..

[89] Zheng H., Zhou Q., Du Y., Li C., Xu P., Lin L., Xiao J., Gao H. (2018). The hypothalamus as the primary brain region of metabolic abnormalities in APP/PS1 transgenic mouse model of Alzheimer’s disease. Biochim. Biophys. Acta Mol. Basis Dis.

[90] Jakel S., Dimou L. (2017). Glial Cells and Their Function in the Adult Brain: A Journey through the History of Their Ablation. Front. Cell Neurosci..

[91] Harris R.A., Joshi M., Jeoung N.H., Obayashi M. (2005). Overview of the molecular and biochemical basis of branched-chain amino acid catabolism. J. Nutr..

[92] Parrella E., Maxim T., Maialetti F., Zhang L., Wan J., Wei M., Cohen P., Fontana L., Longo V.D. (2013). Protein restriction cycles reduce IGF-1 and phosphorylated Tau, and improve behavioral performance in an Alzheimer’s disease mouse model. Aging Cell.

[93] Gratuze M., Julien J., Morin F., Marette A., Planel E. (2017). Differential effects of voluntary treadmill exercise and caloric restriction on tau pathogenesis in a mouse model of Alzheimer’s disease-like tau pathology fed with Western diet. Prog. Neuropsychopharmacol. Prog. Neuropsychopharmacol. Biol. Psychiatry.

[94] Sanchez-Roman I., Barja G. (2013). Regulation of longevity and oxidative stress by nutritional interventions: Role of methionine restriction. Exp. Gerontol..

[95] Ma L., Wang R., Dong W., Zhao Z. (2018). Caloric restriction can improve learning and memory in C57/BL mice probably via regulation of the AMPK signaling pathway. Exp. Gerontol..

[96] Crane P.K., Walker R., Larson E.B. (2013). Glucose levels and risk of dementia. N. Engl. J. Med..

[97] Ernst A., Sharma A.N., Elased K.M., Guest P.C., Rahmoune H., Bahn S. (2013). Diabetic db/db mice exhibit central nervous system and peripheral molecular alterations as seen in neurological disorders. Transl. Psychiatry.

[98] Kim I., Lee J., Hong H.J., Jung E.S., Ku Y.H., Jeong I.K., Cho Y.M., So I., Park K.S., Mook-Jung I. (2010). A relationship between Alzheimer’s disease and type 2 diabetes mellitus through the measurement of serum amyloid-beta autoantibodies. J. Alzheimers Dis..

[99] Macklin L., Griffith C.M., Cai Y., Rose G.M., Yan X.X., Patrylo P.R. (2017). Glucose tolerance and insulin sensitivity are impaired in APP/PS1 transgenic mice prior to amyloid plaque pathogenesis and cognitive decline. Exp. Gerontol..

[100] Matsuzaki T., Sasaki K., Tanizaki Y., Hata J., Fujimi K., Matsui Y., Sekita A., Suzuki S.O., Kanba S., Kiyohara Y. (2010). Insulin resistance is associated with the pathology of Alzheimer disease: The Hisayama study. Neurology.

[101] Ramos-Rodriguez J.J., Ortiz O., Jimenez-Palomares M., Kay K.R., Berrocoso E., Murillo-Carretero M.I., Perdomo G., Spires-Jones T., Cozar-Castellano I., Lechuga-Sancho A.M. (2013). Differential central pathology and cognitive impairment in pre-diabetic and diabetic mice. Psychoneuroendocrinology.

[102] Takeda S., Sato N., Uchio-Yamada K., Sawada K., Kunieda T., Takeuchi D., Kurinami H., Shinohara M., Rakugi H., Morishita R. (2010). Diabetes-accelerated memory dysfunction via cerebrovascular inflammation and Abeta deposition in an Alzheimer mouse model with diabetes. Proc. Natl. Acad. Sci. USA.

[103] Carrera I., Etcheverria I., Li Y., Fernandez-Novoa L., Lombardi V., Vigo C., Palacios H.H., Benberin V.V., Cacabelos R., Aliev G. (2013). Immunocytochemical Characterization of Alzheimer’s Disease Hallmarks in APP/PS1 Transgenic Mice Treated with a New Anti-Amyloid-beta Vaccine. Cent. Asian J. Glob. Health.

[104] Fang Y., Yao L., Li C., Wang J., Chen S., Zhou X.F., Liao H. (2016). The blockage of the Nogo/NgR signal pathway in microglia alleviates the formation of Abeta plaques and tau phosphorylation in APP/PS1 transgenic mice. J. Neuroinflammation.

[105] Devi L., Ohno M. (2010). Phospho-eIF2alpha level is important for determining abilities of BACE1 reduction to rescue cholinergic neurodegeneration and memory defects in 5XFAD mice. PLoS ONE.

[106] Taylor P.M. (2014). Role of amino acid transporters in amino acid sensing. Am. J. Clin. Nutr..

