# Supplementary material for: Branched-Chain Amino Acids Are Linked with Alzheimer’s Disease-Related Pathology and Cognitive Deficits
Source: Cells. 2022 Nov 7;11(21):3523. doi: 10.3390/cells11213523 (PMC9658564; doi:10.3390/cells11213523)
Supplement: Supplementary file 1 [file cells-11-03523-s001.zip › cells-1964977-supplementary.pdf]

Table S1-1

ID	Con / AD	Con / T2D	SEX	Age	BMI	T2D	CVHATT	CVAFIB	CVANGIO	CVBYPASS	CVPACE	CVCHF
55000129	Con	Con	Male	66	25.3	N	0	0	0	0	0	0
61000543	Con	Con	Male	76	32.3	N	0	0	0	0	0	0
25120185	Con	Con	Male	63	29.1	N	0	0	0	0	0	0
25120065	Con	Con	Male	72	26.5	N	0	0	0	0	0	0
55000309	Con	Con	Male	68	22	N	0	0	0	0	0	0
25140074	Con	Con	Male	79	25.9	N	0	0	0	0	0	0
55000625	Con	Con	Male	83	35.9	N	0	0	0	0	0	0
51002848	Con	Con	Male	61	28.9	N	0	0	0	0	0	0
51002808	Con	Con	Male	76	26.4	N	0	0	0	0	0	0
51002751	Con	Con	Male	60	27	N	0	0	0	0	0	0
**Mean+SEM**				**70±3**	**28±1**							
81100215	Con	T2D	Male	63	24	Y	0	0	2	0	0	0
55000116	Con	T2D	Male	67	29.6	Y	0	0	0	0	0	0
55000007	Con	T2D	Male	77	36.6	Y	0	0	2	0	0	0
51002376	Con	T2D	Male	82	27.1	Y	0	0	0	0	0	0
51002864	Con	T2D	Male	71	29.2	Y	0	0	0	0	0	0
55000615	Con	T2D	Male	51	20.7	Y	0	0	0	0	0	0
25140062	Con	T2D	Male	86	25.6	Y	0	0	0	0	0	0
61100272	Con	T2D	Male	58	27.2	Y	0	0	0	0	0	0
61000417	Con	T2D	Male	69	27.1	Y	0	1	0	0	0	0
51002502	Con	T2D	Male	80	28.7	Y	0	0	0	0	0	0
**Mean+SEM**				**70±4**	**28±1**							
25150026	AD	Con	Male	58	21.7	N	0	0	0	0	0	0
55000655	AD	Con	Male	83	28.4	N	0	0	0	0	0	0
51002852	AD	Con	Male	80	24.6	N	0	0	0	0	0	0
25150023	AD	Con	Male	90	31	N	0	0	0	0	0	0
55000060	AD	Con	Male	63	24.5	N	0	0	0	0	0	0
55000029	AD	Con	Male	76	24.3	N	0	0	0	0	0	0
51002845	AD	Con	Male	67	28.1	N	0	0	0	0	0	0
25070109	AD	Con	Male	65	20	N	0	0	0	0	0	0
51002860	AD	Con	Male	58	22.1	N	0	0	0	0	0	0
51002872	AD	Con	Male	85	23.1	N	0	0	0	0	0	0
**Mean+SEM**				**73±4**	**25±1**							
25100065	AD	T2D	Male	77	29.4	Y	0	0	0	0	0	0
25160004	AD	T2D	Male	71	31	Y	0	0	0	0	0	0
25130117	AD	T2D	Male	74	28.1	Y	0	0	0	0	0	0
25040049	AD	T2D	Male	91	22.3	Y	0	1	2	0	0	0
81100007	AD	T2D	Male	82	22.5	Y	0	0	0	0	0	0
55000392	AD	T2D	Male	86	25	Y	0	0	0	0	0	0
61000520	AD	T2D	Male	67	31.9	Y	0	1	0	2	0	0
81200103	AD	T2D	Male	80	29.1	Y	0	0	0	0	0	0
55000370	AD	T2D	Male	65	28.5	Y	0	0	0	0	0	0
55000575	AD	T2D	Male	81	27.4	Y	0	0	0	0	0	0
**Mean+SEM**				**77±3**	**28±1**							

Subject characteristics

0 = Absent: not indicated by information from medical records or observation
1 = Recent/active: happened within last year or requires active management
2 = Inactive: occurred greater than one year ago without any current therapy

* No statistically significant differences of age or BMI between groups

Table S1-2

ID	Con / AD	Con / T2D	CVOTHR	CBSTROKE	CBTIA	CBOTHR	PD	PDOTHR	SEIZURES	TRAUMBR
55000129	Con	Con	0	0	0	0	0	0	0	0
61000543	Con	Con	0	0	0	0	0	0	0	0
25120185	Con	Con	0	0	0	0	0	0	0	0
25120065	Con	Con	0	0	0	0	0	0	0	0
55000309	Con	Con	0	0	0	0	0	0	0	0
25140074	Con	Con	0	0	0	0	0	0	0	0
55000625	Con	Con	0	0	0	0	0	0	0	0
51002848	Con	Con	0	0	0	0	0	0	0	0
51002808	Con	Con	0	0	0	0	0	0	0	0
51002751	Con	Con	0	0	0	0	0	0	0	0
81100215	Con	T2D	0	0	0	0	0	0	0	0
55000116	Con	T2D	0	0	0	0	0	0	0	0
55000007	Con	T2D	0	0	0	0	0	0	0	0
51002376	Con	T2D	0	0	0	0	0	0	0	0
51002864	Con	T2D	0	0	0	0	0	0	0	0
55000615	Con	T2D	0	0	0	0	0	0	0	0
25140062	Con	T2D	0	0	0	0	0	0	0	0
61100272	Con	T2D	0	0	0	0	0	0	0	0
61000417	Con	T2D	0	0	0	0	0	0	0	0
51002502	Con	T2D	0	0	0	0	0	0	0	0
25150026	AD	Con	0	0	0	0	0	0	0	0
55000655	AD	Con	0	0	0	0	0	0	0	0
51002852	AD	Con	0	0	0	0	0	0	0	0
25150023	AD	Con	0	0	0	0	0	0	0	0
55000060	AD	Con	0	0	0	0	0	0	0	0
55000029	AD	Con	0	0	0	0	0	0	0	0
51002845	AD	Con	0	0	0	0	0	0	0	0
25070109	AD	Con	0	0	0	0	0	0	0	0
51002860	AD	Con	0	0	0	0	0	0	0	0
51002872	AD	Con	0	0	0	0	0	0	0	0
25100065	AD	T2D	0	0	0	0	0	1	0	0
25160004	AD	T2D	0	0	0	0	0	0	0	0
25130117	AD	T2D	0	0	0	0	0	0	0	0
25040049	AD	T2D	0	0	0	0	0	0	0	0
81100007	AD	T2D	0	0	0	0	0	0	0	0
55000392	AD	T2D	0	0	2	0	0	0	0	0
61000520	AD	T2D	0	0	0	0	0	0	0	0
81200103	AD	T2D	0	0	0	0	0	0	0	0
55000370	AD	T2D	0	0	0	0	0	0	0	0
55000575	AD	T2D	0	0	0	0	0	0	0	0

Table S1-3

ID	Con / AD	Con / T2D	HYPERTEN	HYPERCHO	B12DEF	THYROID	INCONTU	INCONTF	CANCER
55000129	Con	Con	1	0	0	0	0	0	0
61000543	Con	Con	0	0	0	0	0	0	0
25120185	Con	Con	1	0	0	0	0	0	0
25120065	Con	Con	1	0	0	0	0	0	0
55000309	Con	Con	1	0	0	0	0	0	0
25140074	Con	Con	0	0	0	0	0	0	0
55000625	Con	Con	1	0	0	0	0	0	0
51002848	Con	Con	1	0	0	0	0	0	0
51002808	Con	Con	1	0	0	0	0	0	0
51002751	Con	Con	0	0	0	0	0	0	0
81100215	Con	T2D	1	1	0	0	0	0	0
55000116	Con	T2D	1	1	0	0	0	0	0
55000007	Con	T2D	1	1	2	0	0	0	0
51002376	Con	T2D	1	1	0	0	0	0	0
51002864	Con	T2D	1	1	0	0	0	0	0
55000615	Con	T2D	1	1	0	0	0	0	0
25140062	Con	T2D	1	1	0	0	0	0	0
61100272	Con	T2D	0	0	0	0	0	0	
61000417	Con	T2D	1	1	0	0	0	0	0
51002502	Con	T2D	1	1	0	0	0	0	0
25150026	AD	Con	0	0	0	0	0	0	0
55000655	AD	Con	1	0	0	0	0	0	0
51002852	AD	Con	1	0	0	0	0	0	0
25150023	AD	Con	1	0	0	0	1	0	0
55000060	AD	Con	0	0	0	0	2	0	0
55000029	AD	Con	0	0	0	0	0	0	0
51002845	AD	Con	0	0	0	0	0	0	0
25070109	AD	Con	2	0	0	0	0	0	0
51002860	AD	Con	0	0	0	0	0	0	0
51002872	AD	Con	0	0	0	0	0	0	0
25100065	AD	T2D	1	1	0	0	1	1	0
25160004	AD	T2D	0	0	0	0	0	0	0
25130117	AD	T2D	0	1	0	2	1	1	0
25040049	AD	T2D	0	1	1	0	1	0	0
81100007	AD	T2D	1	1	0	0	0	0	
55000392	AD	T2D	0	1	2	0	1	1	0
61000520	AD	T2D	1	1	0	0	1	1	0
81200103	AD	T2D	1	1	0	0	0	0	0
55000370	AD	T2D	0	0	0	0	0	0	0
55000575	AD	T2D	1	1	0	0	0	0	0

Table S1-4

ID	Con / AD	Con / T2D	DEP2YRS	DEPOTHR	ALCOHOL	TOBACLstYr	ABUSOTHR	PSYCDIS	IBD
55000129	Con	Con	0	0	0	0	0	0	0
61000543	Con	Con	0	0	0	0	0	0	0
25120185	Con	Con	0	0	0	0	0	0	0
25120065	Con	Con	0	0	0	1	0	0	0
55000309	Con	Con	0	0	0	0	0	0	0
25140074	Con	Con	0	0	0	0	0	0	0
55000625	Con	Con	0	0	0	0	0	0	0
51002848	Con	Con	0	0	0		0	0	0
51002808	Con	Con	0	0	0	0	0	0	0
51002751	Con	Con	0	0	0	0	0	0	0
81100215	Con	T2D	0	0	0		0	0	0
55000116	Con	T2D	1	1	0	0	0	0	0
55000007	Con	T2D	0	0	0	0	0	0	0
51002376	Con	T2D	0	0	0	0	0	0	0
51002864	Con	T2D	0	0	0		0	0	0
55000615	Con	T2D	0	0	0	0	0	0	0
25140062	Con	T2D	0	0	0	0	0	0	0
61100272	Con	T2D	0	0	0		0	0	
61000417	Con	T2D	0	0	0		0	0	0
51002502	Con	T2D	0	0	0	0	0	0	0
25150026	AD	Con	0	0	0	0	0	0	0
55000655	AD	Con	0	0	0	0	0	0	0
51002852	AD	Con	0	0	0	0	0	0	0
25150023	AD	Con	1	0	0	0	0	0	0
55000060	AD	Con	1	0	0	0	0	0	0
55000029	AD	Con	1	0	0	0	0	0	0
51002845	AD	Con	0	0	0		0	0	0
25070109	AD	Con	0	1	0	0	0	0	0
51002860	AD	Con	0	0	0	1	0	0	0
51002872	AD	Con	0	0	0	0	0	0	0
25100065	AD	T2D	1	1	0	0	0	0	0
25160004	AD	T2D	1	1	0	0	0	0	0
25130117	AD	T2D	1	0	2	0	0	0	0
25040049	AD	T2D	0	0	0	0	0	0	0
81100007	AD	T2D	0	0	0		0	0	
55000392	AD	T2D	0	0	0	0	0	0	0
61000520	AD	T2D	1	1	0		0	1	2
81200103	AD	T2D	0	1	0	0	0	0	0
55000370	AD	T2D	0	0	0		0	0	0
55000575	AD	T2D	0	0	0	0	0	0	0

Table S1-5

ID	Con / AD	Con / T2D	Arthritic	AutoImm	Chron_Oth
55000129	Con	Con	0	0	0
61000543	Con	Con	0	0	0
25120185	Con	Con	0	0	0
25120065	Con	Con	0	0	0
55000309	Con	Con	0	0	0
25140074	Con	Con	0	0	0
55000625	Con	Con	0	0	0
51002848	Con	Con	0	0	0
51002808	Con	Con	0	0	0
51002751	Con	Con	0	0	0
81100215	Con	T2D	0	0	0
55000116	Con	T2D	0	0	0
55000007	Con	T2D	0	0	0
51002376	Con	T2D	0	0	0
51002864	Con	T2D	0	0	0
55000615	Con	T2D	0	0	0
25140062	Con	T2D	0	0	0
61100272	Con	T2D			
61000417	Con	T2D	0	0	0
51002502	Con	T2D	0	0	0
25150026	AD	Con	0	0	0
55000655	AD	Con	0	0	0
51002852	AD	Con	0	0	0
25150023	AD	Con	1	0	0
55000060	AD	Con	0	0	0
55000029	AD	Con	0	0	0
51002845	AD	Con	0	0	0
25070109	AD	Con	0	0	0
51002860	AD	Con	0	0	0
51002872	AD	Con	0	0	0
25100065	AD	T2D	0	0	0
25160004	AD	T2D	0	0	0
25130117	AD	T2D	1	0	0
25040049	AD	T2D	0	0	0
81100007	AD	T2D			
55000392	AD	T2D	0	0	0
61000520	AD	T2D	1	0	0
81200103	AD	T2D	0	0	1
55000370	AD	T2D	0	0	0
55000575	AD	T2D	0	0	0

CVHATT: Heart attack
CVAFIB: Atrial fibrillation
CVANGIO: Angioplasty/endarterectomy/stent
CVBYPASS: Cardiac bypass procedure
CVPACE: Pacemaker
CVCHF: Congestive heart failure
CVOTHR: Other heart diseases
HYPERTEN: Hypertension
HYPERCHO: Hypercholesterolemia
B12DEF: B12 deficiency
THYROID: Thyroid disease
INCONTU: Incontinence – urinary
INCONTF: Incontinence – bowel
CBSTROKE: Stroke
CBTIA: Transient ischemic attack
CBOTHR: Other CVDs
PD: Parkinson’s disease
PDOTHR: Other Parkinsonian disorders
SEIZURES: Seizures
TRAUMBR: Traumatic brain injury
DEP2YRS: Depression active in last 2 years
DEPOTHR: Depression before last 2 years
ALCOHOL: Alcohol abuse
TOBACLstYr: Cigarette smoking in last year
PSYCDIS: Psychiatric disorders
IBD: Inflammatory bowel disease

Table S2-1

Name	Ratio (AD/C)	Log2 FC	P value
Undecanedioic acid	1.66	0.73	0.007
Pyridoxamine;2-Methyl-4-aminomethyl-5-hydroxymethyl-3-	0.67	-0.57	0.001
Piperidine	0.49	-1.04	0.019
Phenyl sulfoxide	0.74	-0.43	0.023
PEG n6	1.12	0.16	0.010
Osthol	0.70	-0.50	0.055
N,N-Diisopropylethylamine (DIPEA)	0.59	-0.76	0.001
Militarinone A	1.53	0.61	0.013
metominostrobin	0.69	-0.53	0.025
Mercaptoethanol	0.27	-1.87	0.000
Meloxicam	0.28	-1.85	0.026
L-Phenylalanine	2.85	1.51	0.000
L-Cystine	0.71	-0.50	0.004
L-Cysteinylglycine disulfide	0.65	-0.62	0.043
indol-2-one	0.37	-1.44	0.014
Hypoxanthine	1.47	0.55	0.011
Hydroxymethylphosphonate	1.56	0.64	0.014
Hydroxymethylphosphonate	1.59	0.67	0.000
Hexylbenzene	2.05	1.04	0.051
heptadecenoic acid	3.34	1.74	0.000
Gly-Phe	1.87	0.90	0.019
frescolat ML	0.63	-0.66	0.006

Table S2-2

Name	Ratio (AD/C)	Log2 FC	P value
Fluorene	125.00	6.97	0.000
Fasoracetam	0.22	-2.22	0.000
Ethyl docosahexaenoate	1.98	0.99	0.035
Docosahexaenoic acid methyl ester	0.65	-0.63	0.022
Docosahexaenoic acid ethyl ester	1.70	0.77	0.030
Cyclo(phenylalanyl-prolyl)	0.19	-2.36	0.000
Cyclo(leucylprolyl)	0.11	-3.18	0.000
Cuminaldehyde	2.27	1.18	0.006
Choline	1.43	0.51	0.002
chavicol	0.77	-0.37	0.040
Capsi-amide	0.47	-1.10	0.000
Biliverdin	0.79	-0.34	0.026
Atenolol acid	22.73	4.51	0.033
Arachidonic acid	1.47	0.55	0.000
Acetyl-L-carnitine	0.85	-0.24	0.025
6-Methylnicotinamide	0.11	-3.14	0.000
5-(1-Hydroxyethyl)-3-(2-hydroxypropyl)-2(5H)-furanone	0.45	-1.16	0.043
4-Nitroaniline	0.52	-0.95	0.022
3-Oxo-5beta-cholanate	1.60	0.68	0.039
3-Methoxyflavone	0.04	-4.71	0.000
3-Methoxyflavone	0.04	-4.50	0.000
3-Hydroxysebacic acid	1.61	0.69	0.031

Table S2-3

Name	Ratio (AD/C)	Log2 FC	P value
3,4-Dimethylbenzoic acid	1.63	0.70	0.034
3,4-Dimethylbenzoic acid	0.45	-1.15	0.038
2-Phenylethanol	0.45	-1.16	0.042
2-Oxo-delta3-4_5_5-trimethylcyclopentenylacetate	1.62	0.69	0.035
2-Oxo-5-pentyltetrahydro-3-furancarboxylic acid	0.39	-1.36	0.024
2'-N-Acetylparomamine	0.63	-0.67	0.006
2-Methylthiazolidine	1.19	0.25	0.040
2-linoleoyl-sn-glycero-3-phosphoethanolamine	1.33	0.42	0.010
2-Hydroxycinnamic acid	0.01	-7.56	0.001
Tolmetin	2.33	1.22	0.000
2-Decylfuran	1.26	0.34	0.031
2_2'-Iminodipropanoate	0.69	-0.53	0.013
Sulfamethoxazole	2.92	1.54	0.047
2-(5-Hydroxy-2,6-dioxo-3-piperidinyl)-1H-isoindole-1,3(2H)-	0.07	-3.87	0.000
1-Oleoylglycerophosphocholine	1.41	0.50	0.050
10-Oxodecanoate	0.58	-0.79	0.030
1-[3-Hydroxy-2-(2-hydroxy-2-propanyl)-2,3-dihydro-1-benzofuran-	0.54	-0.89	0.044
1,3,5-Heptatriene	0.43	-1.23	0.041
[SThydroxy(2:0)]17_21-dihydroxypregn-4-ene-3_20-dione	5.95	2.57	0.010

Figure S1

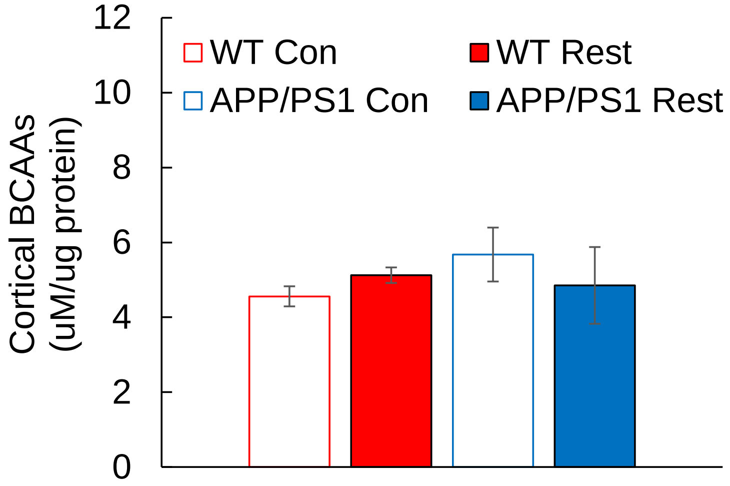

12
10
8
6
4
2
0
Cortical BCAAs (uM/ug protein)
WT Con
WT Rest
APP/PS1 Con
APP/PS1 Rest

Figure S2

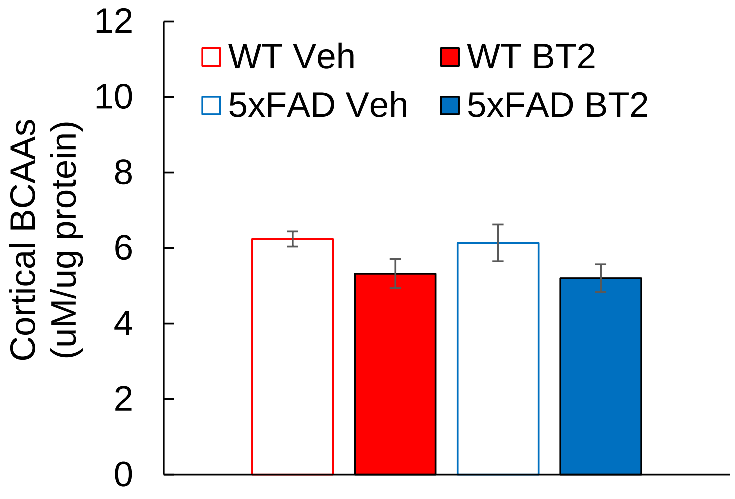

Cortical BCAAs (uM/ug protein)
12
10
8
6
4
2
0
WT Veh
WT BT2
5xFAD Veh
5xFAD BT2

Supplemental Table and Figure Legends

Table S1. Patient information and exclusion characteristics in healthy controls (Con+Con), T2D only (Con+T2D), AD only (AD+Con), AD+T2D (AD+T2D).

Subject characteristics:
0 = Absent: not indicated by information from medical records or observation
1 = Recent/active: happened within last year or requires active management
2 = Inactive: occurred greater than one year ago without any current therapy.

Abbreviation of medical conditions examined:

CVHATT: Heart attack
CVAFIB: Atrial fibrillation
CVANGIO: Angioplasty/endarterectomy/stent
CVBYPASS: Cardiac bypass procedure
CVPACE: Pacemaker
CVCHF: Congestive heart failure
CVOTHR: Other heart diseases
HYPERTEN: Hypertension
HYPERCHO: Hypercholesterolemia
B12DEF: B12 deficiency
THYROID: Thyroid disease
INCONTU: Incontinence – urinary
INCONTF: Incontinence – bowel
CBSTROKE: Stroke
CBTIA: Transient ischemic attack
CBOTHR: Other CVDs
PD: Parkinson's disease
PDOTHR: Other Parkinsonian disorders
SEIZURES: Seizures
TRAUMBR: Traumatic brain injury
DEP2YRS: Depression active in last 2 years
DEPOTHR: Depression before last 2 years
ALCOHOL: Alcohol abuse
TOBACLstYr: Cigarette smoking in last year
PSYCDIS: Psychiatric disorders
IBD: Inflammatory bowel disease

Table S2. Fold changes of serum metabolites between AD and age-matched healthy controls ($p<0.05$)

Figure S1. BCAA levels measured in the neocortex of WT and APP/PS1 mice with control diet or BCAA-restricted diet at the end of two months

Figure S2. BCAA levels measured in the neocortex of WT and 5xFAD mice with 30 day treatment of either vehicle or BT2
